# Domain Selection for Gaussian Process Data: An Application to Electrocardiogram Signals

**DOI:** 10.1002/bimj.70018

**Published:** 2024-11-28

**Authors:** Nicolás Hernández, Gabriel Martos

**Affiliations:** ^1^ School of Mathematical Sciences Queen Mary University of London London UK; ^2^ Department of Statistical Science University College London UK; ^3^ Departamento de Matemática y Estadística Universidad Torcuato Di Tella Buenos Aires Argentina

**Keywords:** domain selection, electrocardiogram signals, Gaussian processes, Kullback–Leibler divergence, intervals of local maximum divergence

## Abstract

Gaussian processes and the Kullback–Leibler divergence have been deeply studied in statistics and machine learning. This paper marries these two concepts and introduce the local Kullback–Leibler divergence to learn about intervals where two Gaussian processes differ the most. We address subtleties entailed in the estimation of local divergences and the corresponding interval of local maximum divergence as well. The estimation performance and the numerical efficiency of the proposed method are showcased via a Monte Carlo simulation study. In a medical research context, we assess the potential of the devised tools in the analysis of electrocardiogram signals.

## 1 Introduction

Every day, millions of complex data patterns circulate globally at unprecedented speeds, driven by advances in information technology. This rapid flow of data has created an overwhelming need for sophisticated models and methods to understand and analyze random process data, such as time series or functional data. Electrocardiogram (ECG) signals are an example of such complex data, which is usually structured in the form of curves almost continuously recorded over a grid of discrete time points. From a medical point of view, when a patient is admitted in the emergency room during a cardiac arrest, one of the few pieces of information available to make a diagnosis is an ECG. It is therefore an extremely useful tool for immediate decision making and it can also be helpful in determining the causes and the gravity of a cardiac pathology (Mullainathan and Obermeyer 2022). Nevertheless, the analysis of ECG data faces important challenges in practice, particularly due to its high dimensionality. Therefore, the study of local features of ECG signals plays a key role in medicine (Mullainathan and Obermeyer 2022; Rodríguez et al. 2015; Wang et al. 2013) for at least two important reasons:

(i) Diagnosis: Identifying time intervals during the cardiac cycle where the ECG signal presents atypical patterns is crucial in order to improve the early diagnosis of different cardiac diseases, increasing the patient survival probability during a cardiac episode.
(ii) Causes and effects: Learning time intervals with an anomalous pattern during the cardiac cycle will help to understand the origins and the consequences of different heart diseases.

Taking into account (i) and (ii) above, the main goal of this paper is to learn from ECG data an *interval with a certain length* where the signals corresponding to disease and healthy subjects differ the most. Throughout this paper, we refer to this problem as that of *domain selection* for ECG signals, but the method devised in this paper also applies to other random processes data in a broad sense. We model ECG signals using Gaussian processes (GPs), a versatile and flexible tool for modeling complex signal patterns (Pérez‐Cruz et al. 2013), and introduce the *local* Kullback–Leibler (KL) divergence (Kullback and Leibler 1951) as we formally discussed in Section 2, so to learn intervals of maximum local divergence between GPs. Recent contributions in the related literature explore *domain selection* methods—a.k.a. variable selection in functional data (Baíllo, Cuevas, and Fraiman 2011; Berrendero, Cuevas, and Torrecilla 2016; Pini and Vantini 2017)—to achieve accurate prediction for functional data classification methods and to assess local differences between functional means in a two‐sample problem. Also, in the context of statistics in medicine, the authors in Martos and de Carvalho (2018) propose a Mann–Whitney type of statistic for functional data to learn about intervals at which two processes differ the most, based on aspects related with symmetry. Our approach differs from the ones mentioned above in at least two important ways: (i) Here the ultimate goal is neither to classify nor to test hypothesis with random processes data, but rather to *learn the interval with a given length where two random processes*, which corresponds to groups of ECG signals, *differ the most*; (ii) Our approach relies on GP and the KL divergence, whereas the aforementioned methodologies have mainly been designed in the context of functional data. As a by‐product, we also contribute on the following points:

- Optimization: We introduce the interval of local maximum divergence through a set function optimiation problem. Therefore, the estimation methods proposed in the paper contribute to the literature on set function optimization.
- Classification: When the analysis of ECG signals also entails the discrimination between groups (i.e., healthy vs. disease), our method could benefit other standard functional classifiers if they are applied on a small interval where the two processes differ the most, rather than treating the entire signal domain equally.
- Storage efficiency: If only a subset of the entire domain is found to be relevant in order to assess differences between healthy subjects and disease patients, then this suggests the potential benefit for collecting and saving only a smaller subset of ECG signals.
- Miscellany: In the paper, we also establish conditions for the existence of an interval of local maximum divergence; consider subtleties entailed in the estimation of local KL divergences and the interval of local maximum divergence; and also discuss variants and extensions around the proposed methodology.

The reminder of the paper is organized as follows: In Section 2, we introduce the local KL divergence for GP and the interval of local maximum divergence, and also discuss suitable corresponding estimation methods. In Section 3, we present Monte Carlo evidence to assess the consistency of our estimator, while in Section 4, we illustrate the method with an ECG signals data application. Finally, in Section 5, we discuss the results and conclude our work.

## 2 Materials and Methods

The goal in this section is to introduce the probabilistic framework to assess local differences between GPs. To this end, let $X∼N(\mu_{X},\sigma_{X})$ and $Y∼N(\mu_{Y},\sigma_{Y})$ be two normally distributed independent random variables, where $\mu_{\ell }$ and $\sigma_{\ell }$ for ℓ∈{X,Y} denotes the corresponding mean and variance parameters; then the KL divergence between these normally distributed random variables can be written in closed from:

(1) $$\text{KL}\left(X||Y\right)=\frac{1}{2}\left(\frac{\sigma_{X}^{2}}{\sigma_{Y}^{2}}-1+\frac{{\left(\mu_{X}-\mu_{Y}\right)}^{2}}{\sigma_{Y}^{2}}+\ln\left(\frac{\sigma_{Y}^{2}}{\sigma_{X}^{2}}\right)\right).$$

The divergence in Equation (1) is a functional that quantifies the dissimilarity in the distribution of two Gaussian random variables X and Y; and more importantly, Equation (1) is easy and computationally cheap to evaluate when X and Y are multivariate normal random vectors. Next, we discuss how to extrapolate Equation (1) to compute intervals of local maximum divergence for GPs.

### 2.1 Local Kullback–Leibler Divergence for Gaussian Processes

Let $X\left(t\right)∼GP(\mu_{X}\left(t\right),\sigma_{X}\left(t,s\right))$ be a GP with mean function $\mu_{X}\left(t\right)=E\left\{X\left(t\right)\right\}$ and variance function $\sigma_{X}\left(t,s\right)=E\left\{\left(X\left(t\right)-\mu_{X}\left(t\right)\right)\left(X\left(s\right)-\mu_{X}\left(s\right)\right)\right\}$, indexed on the compact set T⊂R; we address the following problem on the comparison of two GP:

**Learning Problem**. *Given data drawn from GPs* $X\left(t\right)∼GP(\mu_{X}\left(t\right),\sigma_{X}\left(t,s\right))$ *and* $Y\left(t\right)∼GP(\mu_{Y}\left(t\right),\sigma_{Y}\left(t,s\right))$ *, indexed on the same compact domain* T⊂R *, learn the interval with a given certain length where the two processes statistically differ the most*.

Since the mean and variance functions completely determine the law of X(t) and Y(t); hereafter, we assume that there exists a compact set A with λ(A)>0, and another set B with λ(B)>0, where λ(·) is the Lebesgue measure of the sets A and B, and positive constant ν, such that one of the following scenarios of local differences holds:

(A) For all t∈A⊆T: $|\mu_{X}\left(t\right)-\mu_{Y}\left(t\right)|>\nu$, while for $t^{\prime }\notin A$ it holds that $|\mu_{X}\left(t^{\prime }\right)-\mu_{Y}\left(t^{\prime }\right)|\leq \nu$.
(B) For all (t,s)∈B⊆T×T: $|\sigma_{X}\left(t,s\right)-\sigma_{Y}\left(t,s\right)|>\nu$, while $|\sigma_{X}\left(t^{\prime },s^{\prime }\right)-\sigma_{Y}\left(t^{\prime },s^{\prime }\right)|\leq \nu$ if $(t^{\prime },s^{\prime })\notin B$.
(C) Local mean and variance differences correspond to scenarios (A) and (B) simultaneously, possibly on different subsets $A_{\mu }$ and $B_{\sigma }$ and for different thresholds constant $\nu_{\mu }$ and $\nu_{\sigma }$.

These three scenarios were considered taking into account the expression of the KL—see Equation (1), which is defined using the first and second moments of the distributions: In Equation (1), one can identify the midterm $\frac{{\left(\mu_{X}-\mu_{Y}\right)}^{2}}{\sigma_{Y}^{2}}$, which takes into account the differences between the mean vectors, motivating scenario (A). This means that if the second‐order moments of the processes are identical, this is the only term that will prevail in the KL divergence. On the other hand, the first term, $\frac{\sigma_{X}^{2}}{\sigma_{Y}^{2}}$, and the last term, $\ln\left(\frac{\sigma_{Y}^{2}}{\sigma_{X}^{2}}\right)$, motivate scenario (B). Therefore, when the first‐order moments of the processes are identical, these terms will define the KL divergence. Scenario (C) is motivated then by a combination of the previous settings.

**FIGURE 1:**
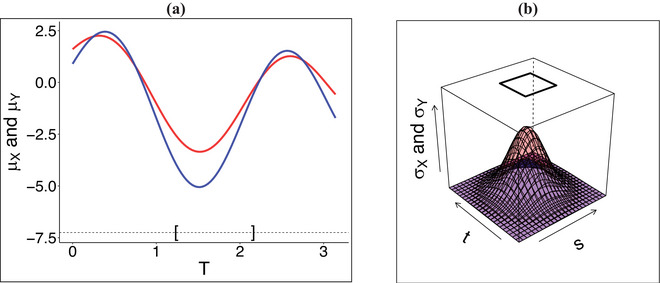
(a) Mean functions $\mu_{X}\left(t\right)$ and $\mu_{Y}\left(t\right)$ represented with solid red (—) and blue (—) lines, respectively. (b) Variance functions $\sigma_{X}\left(t,s\right)$ and $\sigma_{Y}\left(t,s\right)$ in transparent red ( • ) and blue ( • ) surfaces, respectively. Length 1 interval of local maximum divergence depicted with brackets in (a) and a black square in (b).

Scenario A is illustrated in Figure 1(a): Solid lines represent the mean functions of two GP; notice that over a relatively small time interval—around the time point t=1.5—the difference $|\mu_{X}\left(t\right)-\mu_{Y}\left(t\right)|$ exceeds a certain threshold $\nu_{\mu }$. In Figure 1(b), we depict scenario B: The colored surfaces represent the covariance functions of two GP; where $\sigma_{Y}\left(s,t\right)=\delta \sigma_{X}\left(s,t\right)$ and δ<1 is a tilting parameter. Notice that over a relatively small time interval—highlighted with a black square on the top—the difference $|\sigma_{X}\left(t,s\right)-\sigma_{Y}\left(t,s\right)|$ is above a certain threshold $\nu_{\sigma }$. Scenario C corresponds to Figure 1(a) and (b) simultaneously.

The goal of this paper is to develop a local KL divergence measure and related estimation methods to learn about intervals or regions, such as the one showed between brackets in Figure 1(a) and the black square in Figure 1(b), where two GPs differ the most (i.e., where the functional parameters are the most dissimilar). To this end, it will be technically convenient and computationally efficient to use a finite‐dimensional (or discrete) representation of GP data. Without loss of generality, we assume that data are recorded and stored over the same discrete and equally spaced grid of points $T=(t_{1},\cdots ,t_{p})\subset T$, being p≫0 (other more general cases are easy to tackle as we discuss in Section 3). In this setting, GP data correspond to realizations of p‐variate Gaussian random vectors $X_{T}\equiv \left(X\left(t_{1}\right),\cdots ,X\left(t_{p}\right)\right)∼N_{p}\left(\mu_{T,X},\Sigma_{T,X}\right)$ and $Y_{T}\equiv \left(Y\left(t_{1}\right),\cdots ,Y\left(t_{p}\right)\right)∼N_{p}\left(\mu_{T,Y},\Sigma_{T,Y}\right)$ where $\mu_{T,X}=\left(\mu_{X}\left(t_{1}\right),\cdots ,\mu_{X}\left(t_{p}\right)\right)$ and $\mu_{T,Y}=\left(\mu_{Y}\left(t_{1}\right),\cdots ,\mu_{Y}\left(t_{p}\right)\right)$ are the corresponding means, and $\Sigma_{T,X}\in R^{p\times p}$ and $\Sigma_{T,Y}\in R^{p\times p}$ the corresponding p×p positive‐definite (PD) variance matrices (i.e., ${\left[\Sigma_{T,X}\right]}_{ij}=\sigma_{X}\left(t_{i},t_{j}\right)$ and ${\left[\Sigma_{T,Y}\right]}_{ij}=\sigma_{Y}\left(t_{i},t_{j}\right)$ for i=1,⋯,p and j=1,⋯,p).

Under this high‐dimensional GP's representation, the KL divergence (Kullback and Leibler 1951) is a natural metric to assess differences in distribution between two GP's. The KL divergence between GP X and Y over the grid T is computed as follows (Pardo 2005):

(2) $${\text{KL}}_{T}\left(X||Y\right)\equiv \frac{1}{2}\left(\mathrm{tr}\left(\Sigma_{T,Y}^{-1}\Sigma_{T,X}-I_{p}\right)+\Delta_{T}^{T}\Sigma_{T,Y}^{-1}\Delta_{T}+\ln\left(\frac{\det\Sigma_{T,Y}}{\det\Sigma_{T,X}}\right)\right),$$

where $\Delta_{T}=\left(\mu_{T,Y}-\mu_{T,X}\right)$, tr(Σ) denote the trace of Σ, and det(Σ) the determinant of Σ. Some comments on the KL divergence are in order: (i) The expression in Equation (2) is a generalization of the corresponding univariate KL divergence in Equation (1). (ii) The KL divergence is not symmetric; nevertheless, the symmetrization is straightforward: consider, for instance, ${\text{KL}}_{T}\left(X||Y\right)/2+{\text{KL}}_{T}\left(Y||X\right)/2$. (iii) Interestingly, the KL divergence considers simultaneously differences in mean and variance, that is, both mean and variance appear together in Equation (2). (iv) In the case of GP with the same covariance function, then $2{\text{KL}}_{T}\left(X||Y\right)=\Delta_{T}^{T}\Sigma_{T,Y}^{-1}\Delta_{T}$ corresponds to the squared Mahalanobis distance (McLachlan 1999) between the two GP.

Our goal is to learn subsets of T where the two processes differ the most; therefore, the KL divergence is a suitable “statistical distance” to assess local differences between GP. For any subset A⊆T, we define the local‐KL divergence as follows:

(3) $${\text{KL}}_{A}\left(X||Y\right)\equiv \frac{1}{2}\left(\mathrm{tr}\left(\Sigma_{A,Y}^{-1}\Sigma_{A,X}-I_{\left|A\right|}\right)+\Delta_{A}^{T}\Sigma_{A,Y}^{-1}\Delta_{A}+\ln\left(\frac{\det\Sigma_{A,Y}}{\det\Sigma_{A,X}}\right)\;\right),$$

where $\Delta_{A}=\left(\mu_{A,Y}-\mu_{A,X}\right)$ and $\left\{\mu_{A},\Sigma_{A}\right\}$ denotes suitable partitions of $\left\{\mu_{T},\Sigma_{T}\right\}$ in correspondence with the subset A⊆T. Local KL divergences for GP's data also have a number of interesting properties which we summarize next (refer to the Appendix for detailed proofs).

Proposition 2.1 ${\text{KL}}_{A}\left(X||Y\right)$ is a set function that satisfies the following properties:

(a) **Nonnegative:** For fixed GPs X and Y, it holds: ${\text{KL}}_{A}\left(X||Y\right):P_{T}\to R_{0}^{+}$ where $P_{T}$ denotes the power set of T. Moreover, ${\text{KL}}_{A}\left(X||Y\right)=0$ if and only if $\mu_{X}\left(t\right)=\mu_{Y}\left(t\right)$ for all t∈A and $\sigma_{X}\left(t,s\right)=\sigma_{Y}\left(t,s\right)$ for all (t,s)∈A×A.
(b) Let $\mu_{X}\left(t\right)$ and $\mu_{Y}\left(t\right)$ be continuous functions in T and let $\sigma_{X}\left(s,t\right)$ and $\sigma_{Y}\left(s,t\right)$ be PD covariance functions, then the local KL divergence is *upper bounded* (i.e., ${\text{KL}}_{T}\left(X||Y)<\infty \right)$ and a *monotone* set function (i.e., for all $A^{\prime }\subseteq A$, it holds ${\text{KL}}_{A^{\prime }}\left(X||Y\right)\leq {\text{KL}}_{A}\left(X||Y\right)$).

Note that ${\text{KL}}_{A}\left(X||Y\right)$ accounts simultaneously for differences in mean and variance between the two processes. We therefore define the most *discriminating subset of points* in T as follows:

Definition 2.1 Let |·| be the counting measure on $P_{T}$, we define the subset ${\tilde{A}}^{*}\left(\tilde{c}\right)\subset T$, for any $1\leq \tilde{c}\leq \left|T\right|$, as the *domain selection* subset that solves the following set function optimization problem:

(4) $$\max_{A\subset T}{\text{KL}}_{A}\left(X||Y),\;\text{s.t.}\;|A\right|\leq \tilde{c}.$$

Interestingly, this definition resembles some developments in the context of variable selection for functional data classification (Berrendero, Cuevas, and Torrecilla 2016), where the authors propose a maxima‐hunting approach to search for isolated time points in the domain corresponding to random processes X(t) and Y(t) where the covariance distance (Székely, Rizzo, and Bakirov 2007) is maximal. Our approach is somehow similar in the sense that we maximize a metric that accounts for local differences between the processes, but differs in two important ways: (i) we work under the GP assumption in order to rely on an easy‐to‐compute metric that accounts for differences in mean and variance simultaneously; and (ii) our main goal is to learn about the local maximum divergence interval. Some additional comments are in order: First, the discrete set ${\tilde{A}}^{*}\left(\tilde{c}\right)$ does not necessarily correspond to an interval; and second, Equation (4) entails a cumbersome combinatorial set function optimization problem even for moderate values of p. Since the goal of the paper is to select a subset of the domain of the GPs instead of isolated points, we put some additional structure on the *shape* of our candidate set $A^{*}$ as in the following definition.

Definition 2.2 Consider $C_{T}$ as the collection of all contiguous subsets from the ground set T, such that for any $A\in C_{T}$, then $A=\{t_{k},t_{k+1},\cdots t_{k+l}\}$ for some 1≤k≤p and 0≤l≤p−k. Then, the interval of local maximum KL divergence of size c∈(0,1), denoted onward as $A^{*}\left(c\right)$, is defined throughout the following set function optimization problem:

(5) $$\max_{A\in C_{T}}{\text{KL}}_{A}\left(X||Y\right),\;\text{s.t. len}\left(A\right)\leq c\lambda \left(T\right),$$

where len(A)=|A|−1 is the length function corresponding to subset A.

The parameter c∈(0,1) in Equation (5) determines the proportion of the domain to be selected and avoids the use of a particular length scale as is the case of $\tilde{c}$ in Equation (4). The determination of the interval length, c, is data‐driven. This allows practitioners to employ various methodologies for its selection. One approach involves utilizing cross‐validation, where the data set is partitioned into training and testing sets, allowing for the assessment of different interval lengths based on predictive performance. Another strategy is to employ an elbow function, analogous to a scree plot in principal component analysis (PCA), by computing divergences for different lengths and identifying the point where additional length ceases to significantly enhance information capture.

The existence of $A^{*}\left(c\right)$ follows from the result stated in Proposition 2.1(b) and the fact that $P_{T}$ is finite (see the Appendix for further details). However, the interval of local maximum divergence $A^{*}\left(c\right)$ does not need to be unique, as can be easily seen by considering the limiting case where GP's X(t) and Y(t) have the same mean and variance functions. In such a case, every compact subset of T with measure c is a set of maximum KL local divergence. Moreover, one can learn several disjoints intervals of maximum KL local divergence by applying sequential analysis. This involves selecting a new interval of maximum KL local divergence, after removing previously selected intervals. It becomes crucial then to ensure that the length of this new interval aligns with the remaining available domain.

### 2.2 Learning Intervals With Local Maximum KL Divergence From Data

To learn $A^{*}\left(c\right)$ from data, we consider samples recorded over the same discrete grid T: $D_{X}={\left\{x_{i}\right\}}_{i=1}^{n}$ and $D_{Y}={\left\{y_{j}\right\}}_{j=1}^{m}$ drawn from $GP(\mu_{X}\left(t\right),\sigma_{X}\left(t,s\right))$ and $GP(\mu_{Y}\left(t\right),\sigma_{Y}\left(t,s\right))$, respectively (more general sampling designs are discussed in § 2.2). Let $\Psi_{T,X}=\left\{\mu_{T,X},\Sigma_{T,X}\right\}$ be the true parameters corresponding to GP X(t), and the maximum likelihood estimates are given by:

$${\hat{\Psi }}_{T,X}=\left\{{\hat{\mu }}_{T,X}=\frac{1}{n}\sum_{i=1}^{n}x_{i};\;{\hat{\Sigma }}_{T,X}=\frac{1}{n}\sum_{i=1}^{n}\left(x_{i}-{\hat{\mu }}_{T,X}\right){\left(x_{i}-{\hat{\mu }}_{T,X}\right)}^{T}\right\},$$

and an analogous expression holds for ${\hat{\Psi }}_{T,Y}$ as well. Plugging suitable partitions ${\hat{\Psi }}_{A,X}$ and ${\hat{\Psi }}_{A,Y}$ corresponding to the subset A into Equation (5) gives us an estimate of ${\hat{\text{KL}}}_{A}\left(X||Y\right)$. Notice that the trace and determinant are continuous functions in the space of real symmetric matrices, then ${\hat{\text{KL}}}_{A}\left(X||Y\right)$ inherits interesting statistical properties, in particular for a fixed p, consistency, asymptotic normality, and efficiency, providing that p/n→0, p/m→0, and m/n→ρ∈(0,1) as n→∞ and m→∞. Having in hand a consistent estimator of the local KL divergence, to learn about $A^{*}\left(c\right)$, for c∈(0,1), we follow the step provided in Algorithm 1.

ALGORITHM 1 **Estimating the interval of local maximum KL divergence from GP data.**

**Table:** 

**Inputs:** $D_{X}$ and $D_{Y}$ and a length constrain 0<c≤1.
**Step 1:** Parameter estimation.
IF: $D_{X}$ and $D_{Y}$ are recorded over the same grid T, compute Maximum Likelihood estimates ${\hat{\Psi }}_{T,X}$ and ${\hat{\Psi }}_{T,Y}$ from data.
ELSE: Use your favorite (semi/non) parametric estimation method to compute ${\hat{\mu }}_{\ell }\left(t\right)$ and ${\hat{\sigma }}_{\ell }\left(t,s\right)$ from $D_{\ell }$ for ℓ={X,Y}.
**Step 2:** Exhaustive optimization: Let $C_{T}\left(c\right)$ be the set of all contiguous subsets in T such that len(A)≤cλ(T), then for all $A\in C_{T}\left(c\right)$ compute ${\hat{\text{KL}}}_{A}\left(X||Y\right)$ and return: ${\hat{A}}_{c}^{*}\equiv {arg max}_{A\in C_{T}\left(c\right)}{\hat{\text{KL}}}_{A}\left(X||Y\right)$.

Algorithm 1 warrants some comments: Step 1 is carried only once even in the case of estimating several intervals of local maximum KL divergence for different values of c. For the computation of the maximum likelihood estimates ${\hat{\Psi }}_{T,X}$ and ${\hat{\Psi }}_{T,Y}$, it is necessary to define a common grid T so to evaluate $\left({\hat{\mu }}_{\ell }\left(t\right),{\hat{\sigma }}_{\ell }\left(t,s\right)\right)$ over T for ℓ={X,Y}, respectively.

In our R implementation, the set function optimization problem in Step 2 is solved via the evaluation of ${\hat{\text{KL}}}_{A}\left(X||Y\right)$ over all sets in $C_{T}\left(c\right)$; nevertheless, other more efficient derivative‐free optimization approaches (Nocedal and Wright 1999) can be considered as well. As a general comment, our proposed algorithm can also be identified as a *sliding window approach*. In Section 3, we assess the computational efficiency of the estimation method entailed in Algorithm 1 over different data‐generating scenarios.

**The curse of dimensionality**. In the context of GP data, usually, n and m are relatively small in comparison to p=|T|, being often the case where p≫max{n,m}. Since the estimation of $A^{*}\left(c\right)$ depends on the estimation of two p×p covariance matrices, some remedy actions are necessary in order to obtain suitable estimations for $\Sigma_{T,X}$ and $\Sigma_{T,Y}$ from data whenever p is relatively large in comparison to sample sizes n and m. A well‐known strategy is to impose structure in the GP's covariance functions, assuming, for instance, that both processes are conditionally independent in time (i.e., $\sigma_{X}\left(t,s\right)=\sigma_{Y}\left(t,s\right)=0$ for all t≠s), which corresponds to assume that $\Sigma_{T,X}$ and $\Sigma_{T,Y}$ are diagonal covariance matrices. Another less contrived approach to circumvent the curse of dimensionality is to consider a penalized likelihood covariance matrix estimator as follows:

$${\hat{\Sigma }}_{\eta }=\eta \;\hat{\Sigma }+\left(1-\eta \right)\;\text{diag}\left(\hat{\Sigma }\right),\;\text{for}\;\eta \in \left[0,1\right],$$

where η is a regularization parameter that shrinks the maximum likelihood estimator of the variance matrix toward its diagonal (Hastie et al. 2009). The value of η is typically determined using cross‐validation methods. Nevertheless, other approaches such as banding, tapering, and alternative thresholding methods are also available, we refer to (Pourahmadi 2013, Ch. 6) and references therein for more details.

**Sampling designs and misalignments**. In order to simplify the exposition, in Section 2.1, we assume that X(t) and Y(t) are recorded over the same equally space time point grid T, but other sampling designs are also frequent in practice. In such cases where the processes are not recorded over the same grid, to use Algorithm 1, we first need to define a common auxiliary grid T and then proceed as follows:

1. Use a suitable smoothing technique such as kernel smoothing, smoothing splines, or GP regression among others; and estimate the functional parameters $\mu_{\ell }\left(t\right)$ and $\sigma_{\ell }\left(t,s\right)$ for ℓ={X,Y} from data.
2. Consider ${\hat{\mu }}_{\ell }=\left({\hat{\mu }}_{\ell }\left(t_{1}\right)\cdots ,{\hat{\mu }}_{\ell }\left(t_{p}\right)\right)$ and ${\left[\hat{\Sigma }\right]}_{i,j}={\hat{\sigma }}_{\ell }\left(t_{i},t_{j}\right)$, for ℓ={X,Y} and i={1,⋯,p} and j={1,⋯,p} (i.e., the evaluation of the estimated functional parameters over the grid T).

After the estimation of mean vectors and covariance matrices over a common grid T, Step 2 in Algorithm 1 follows straightforwardly. In principle, the auxiliary grid T contains equally spaced time points and has length p according to the resolution defined by the user. Nevertheless, other sampling designs can be considered as well, for instance, sampling T from a multivariate *prior* distribution $\pi_{T}\left(t_{1},\cdots ,t_{p}\right)$. This prior distribution encodes knowledge about the most local divergent interval in T by putting more probability mass over a specific interval A⊂T. This procedure is a natural avenue to study a Bayesian extension of the proposed method to select domain with GP.

GP data are sometimes recorded with different types of random amplitude and time variations that produce a misalignment in data (Ramsay and Li 1998). The GP data asynchrony may act as a confounding factor when the aim is the estimation of the interval of local maximum divergence, since the mean and variance functions estimators defined in § 2.2 are useless. In this case, before the implementation of Step 1 in Algorithm 1, we suggest to preprocess GP data using standard alignment or synchronization tools (a.k.a. as curve registration in functional data) such as the methods described in Berndt and Clifford (1994) and Kazlauskaite, Ek, and Campbell (2019) among others.

**Smoothing**. GP data sometimes present a low signal to noise ratio leading to problems in the estimation of the interval of local maximum divergence. To alleviate low signal‐to‐noise ratio issues, we recommend to smooth GP data using standard methods such as natural splines, or kernel regression, among others (Ullah and Finch 2013). The smoothing process also enables us to use a finer grid of points (see *sampling designs* paragraph). Once the smoothed data are obtained, they can be used as the input in Algorithm 1.

### 2.3 Inference and Prediction

**Inference**. To assess the variability of ${\hat{A}}^{*}\left(c\right)$ as an estimator of $A^{*}\left(c\right)$, we resort to nonparametric bootstrap techniques. For every c∈(0,1), the interval of local maximum divergence and its corresponding estimator can be parametrized in terms of a ball with center $t_{c}$ and radius^1^ $r_{c}$ as follows: $B\left(t_{c}^{*},r_{c}\right)\equiv \left[\min_{t\in A^{*}\left(c\right)}t,\max_{t\in A^{*}\left(c\right)}t\right]$ that corresponds to $A^{*}\left(c\right)$; and $B\left({\hat{t}}_{c}^{*},r_{c}\right)\equiv \left[\min_{t\in {\hat{A}}^{*}\left(c\right)}t,\max_{t\in {\hat{A}}^{*}\left(c\right)}t\right]$ that corresponds to ${\hat{A}}^{*}\left(c\right)$. Then, a 1−α bootstrap confidence interval for $t_{c}^{*}$ is given by ${\text{CI}}_{1-\alpha }\left(t_{c}^{*}\right)\equiv \left[{\hat{t}}_{c,\alpha /2}^{*},{\hat{t}}_{c,1-\alpha /2}^{*}\right]$, where ${\hat{t}}_{c,\alpha }^{*}$ is the α∈(0,1) quantile corresponding to the empirical distribution ${\hat{F}}_{B}\left(t_{c}^{*}\right)$ obtained using B≫0 bootstrap samples from GP data. In the experimental sections, we report ${\hat{F}}_{B}\left(t_{c}^{*}\right)$ and ${\text{CI}}_{1-\alpha }\left(t_{c}^{*}\right)$. Since a confidence interval for a point estimate is a collection of points, then a confidence set for an interval estimate will be a collection of intervals ${\left\{B\left(t,r_{c}\right)\right\}}_{t\in {\text{CI}}_{1-\alpha }\left(t_{c}^{*}\right)}$. To obtain a pictorial representation corresponding to the collections of intervals, for the computation of ${\text{CS}}_{1-\alpha }\left(A^{*}\left(c\right)\right)$, consider:

$${\text{CS}}_{1-\alpha }\left(A^{*}\left(c\right)\right):=\underset{t\in {\text{CI}}_{1-\alpha }\left(t_{c}^{*}\right)}{⋃}B\left(t,r_{c}\right).$$

Some additional comments on the uncertainty quantification are in order. Regardless the experimental design, there is a trade‐off between the value of c and the variability of our estimator. For instance, for p=100 and c=0.1, there are 91 possible intervals in the domain, while for c=0.9, there are only 11 intervals. Hence, the uncertainty associated to ${\hat{A}}^{*}\left(c\right)$ converges to zero as c→1—these correspond to narrower intervals for $t_{c}^{*}$ as c→1. Other confidence interval based on bootstrap procedures can be considered as well, such as the percentile or the Student‐*t* method. Moreover, the parametric bootstrap is also another possible approach, taking into account the Gaussian assumptions. Notice that bootstrap confidence intervals are neither exact nor optimal, but they are largely used in practice since the method is easy to implement and its accuracy is near‐exact. For a general discussion on the asymptotic properties regarding the coverage probability of a bootstrap confidence interval, we refer to Efron and Tibshirani (1994).

**Domain selection and GP classification**. Domain selection is an important preliminary step before training a classification model (Berrendero, Cuevas, and Torrecilla 2016). In the context of GP data, the discriminant analysis (DA) is the Bayes optimal classifier (Fraley and Raftery 2002); hence to classify a new unlabeled instance z—a realization from X(t) or Y(t) recorded over T–, the DA considers the sign of the following discriminant function:

(6) $$\begin{array}{cc} D_{T}\left(z\right) & =\frac{1}{2}\left({\left(z-\mu_{T,Y}\right)}^{T}\Sigma_{T,Y}^{-1}\left(z-\mu_{T,Y}\right)-{\left(z-\mu_{T,X}\right)}^{T}\Sigma_{T,X}^{-1}\left(z-\mu_{T,X}\right)\right.\\ & \;\left.-\ln\left(\frac{\text{det}\;\Sigma_{T,X}}{\text{det}\;\Sigma_{T,Y}}\right)\right)+\ln\left(\frac{\pi_{X}}{\pi_{Y}}\right), \end{array}$$

where $\pi_{X}$ and $\pi_{Y}=1-\pi_{X}$ are the corresponding prior probabilities. $D_{T}\left(z\right)$ encodes the rule to classify z as generated from X(t) or Y(t). In high‐dimensional contexts (p≫0), an important drawback of DA is the lack of reliable estimates of the involved mean vectors and variance matrices. This leads to inaccurate classification results in case no action is taken on the course of dimensionality. In this regard, it will be convenient to use only a small compact subset A⊂T of GP data so to compute the discrimination function. This corresponds to replacing $D_{T}\left(z\right)$ by $D_{A^{*}\left(c\right)}\left(z\right)$ for a suitable value c∈(0,1), reducing the number of parameter entailed in the discrimination analysis. Therefore, choosing a suitable value for c will be crucial to obtain accurate classification results. It is interesting to mention that a choice of $c\;\text{such that}\;\tilde{c}=c\lambda \left(T\right)=1$, would transform the domain selection into a variable selection problem, that is, selecting a variable on the domain of the processes. While this procedure remains valid, it does not allow for the exploitation of the existing information in the covariance matrix of the processes by considering only the variability at the selected variable.

## 3 Simulation Study

In this section, we assess the performance of Algorithm 1 in the estimation of $A^{*}\left(c\right)$ via a Monte Carlo simulation study under three different scenarios in line with the hypothesis stated in § 2.1. To this end, we consider GP data $D_{X}={\left\{x_{i}\right\}}_{i=1}^{n}$ and $D_{Y}={\left\{y_{j}\right\}}_{j=1}^{m}$ in the interval T=[0,π] recorded over a discrete grid T={0π/p,1π/p,⋯,(p−1)π/p}. Following the examples in Figure 1, we set three different scenarios.

**FIGURE 2:**
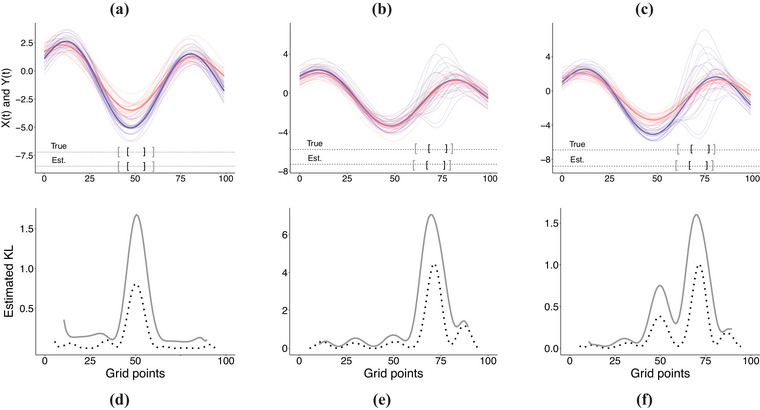
**One‐shot experiment:** Panels (a)–(c) illustrate the realizations of X(t) and Y(t) for scenarios A–C and the corresponding estimated mean functions (depicted in solid red and blue, respectively). Panels (d)–(f) show the estimated local KL divergence for different interval lengths: c=0.1 (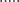) and c=0.2 (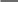); the multiple local maxima in panel (f) corresponds to the particular casuistry of scenario C (for a sufficiently large c, the local divergence curve will be unimodal as well).

**Scenario A: Local differences in mean**. GP data are generated according to the following specification:

$$X\left(t\right)={\left(\beta_{X}+\varepsilon \right)}^{T}\Phi \left(t\right),\;\text{and}\;Y\left(t\right)={\left(\beta_{Y}+\varepsilon \right)}^{T}\Phi \left(t\right),$$

where $\beta_{X}=\left\{1,-2,-1,1,2,-1,2,3,-0.5\right\}$, $\beta_{Y}=\left\{-1,-2,-1,1,2,-1,2,5,-0.5\right\}$, $\Phi \left(t\right)\equiv \{\phi_{1}\left(t\right),\phi_{2}\left(t\right),\cdots ,\phi_{9}\left(t\right)\}$ is a vector functions containing the first nine Fourier basis functions, and ε is a normally distributed random vector $\varepsilon ∼N_{9}\left(0,0.25I_{9}\right)$. For the seek of simplicity, the distribution of ε remains fixed in all scenarios. In this simulation scenario, it holds that $\sigma_{X}\left(t,s\right)=\sigma_{Y}\left(t,s\right)$ for all (t,s)∈T×T; nonetheless, $\mu_{X}\left(t\right)$ is remarkably different to $\mu_{Y}\left(t\right)$ around grid point 50 as can be seen in Figure 2(a).

**Scenario B: Local differences in variance**. Data are generated under the following specification:

$$X\left(t\right)={\left(\beta_{X}+\varepsilon +\gamma e^{-{\left(t-3\pi /4\right)}^{2}}\right)}^{T}\Phi \left(t\right),\;\;\text{and}\;\;Y\left(t\right)={\left(\beta_{Y}+\varepsilon \right)}^{T}\Phi \left(t\right),$$

where $\beta_{X}=\beta_{Y}=\left\{1,-2,-1,1,2,-1,2,3,-0.5\right\}$, and $\gamma ∼N(0,\tau^{2}I_{9})$ is a multivariate normal random vector with $\tau^{2}=1$. Some comments on this scenario are in order: It holds that $\mu_{X}\left(t\right)=\mu_{Y}\left(t\right)$ for all t∈T; nevertheless, $\sigma_{X}\left(t,s\right)$ present more differences with $\sigma_{Y}\left(t,s\right)$ around the grid point 75 (which corresponds to time point t=3π/4) as can be seen in Figure 2(b).

**Scenario C: Local differences in mean and variance**. For this scenario, we consider

$$X\left(t\right)={\left(\beta_{X}+\varepsilon +\gamma e^{-{\left(t-3\pi /4\right)}^{2}}\right)}^{T}\Phi \left(t\right),\;\;\text{and}\;\;Y\left(t\right)={\left(\beta_{Y}+\varepsilon \right)}^{T}\Phi \left(t\right).$$

In this scenario, $\beta_{X}$ and $\beta_{Y}$ take the same values as in scenario A, and γ is defined likewise in scenario B; therefore, it holds that mean and variance functions differ over different subsets in T as can be seen in Figure 2(c).

In Figure 2, panels (a)–(c), we depict one‐shot GP data examples (n=m=25 and p=100) drawn from scenarios A–C, respectively; while in panels (d)–(f), we show the values of the estimated ${\text{KL}}_{A(c)}\left(X||Y\right)$ as a function of the central point of each interval corresponding to A(c) for c={0.10,0.20} (i.e., ${\text{KL}}_{A(c)}\left(X||Y\right)={\text{KL}}_{t_{c}}\left(X||Y\right)$ where $t_{c}$ is the center of the ball $B\left(t_{c},r_{c}\right)=\left[\min_{t\in A(c)}t,\max_{t\in A(c)}t\right]$). Notice that the maximum value of the estimated local divergence in the lower panels in Figures 2(d)–(f) corresponds to an estimated interval of local maximum divergence depicted in the upper panel of Figures 2(a)–(c). In all cases of this one‐shot experiment, the estimated interval of local maximum divergence is close to the true interval of local maximum divergence. To validate the accuracy of the estimation method, we run a Monte Carlo simulation study.

**FIGURE 3:**
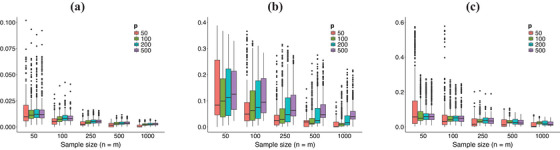
Empirical distribution of AIJD for different sample sizes n, m and grid resolution levels p. Scenarios A‐C in panels (a)–(c), respectively.

**Monte Carlo Results**. The Monte Carlo simulation study considers, in each scenario, M=500 data replicates for sample sizes m=n∈{50,100,250,500,1000} and grid resolution levels p∈{50,100,200,500}. To assess the estimation performance of Algorithm 1, we depict in Figure 3 the empirical distribution of the *a*verage *i*ntegrated *J*accard *d*istance (AIJD) defined as:

(7) $$\text{AIJD}=E\{\int_{0}^{1}\left[1-\frac{|A^{*}\left(c\right)\cap {\hat{A}}^{*}\left(c\right)|}{|A^{*}\left(c\right)\cup {\hat{A}}^{*}\left(c\right)|}\right]dc\}.$$

The *Jaccard* Index (Jaccard 1912) is the natural measure to assess similarity between sets, and we estimate AIJD using the trapezoidal rule over a uniform grid for c. The numerical analysis of our estimation method can be seen in Figure 3. Some comments about the Monte Carlo results are in order. As sample size n and m increase, then the AIJD decreases in all scenarios, and this suggests a consistent estimation method. For scenarios A and C, the numerical experiment shows highly accurate results even for low sample sizes (see, e.g., n=m=50), while in scenario B, the results present more variability. Notice also that for fixed n and m, the estimated AIJD increases in average as p increases (in almost all scenarios) due to the accumulating errors in the estimation of all entries of the covariance p×p matrices as p increases.

**FIGURE 4:**
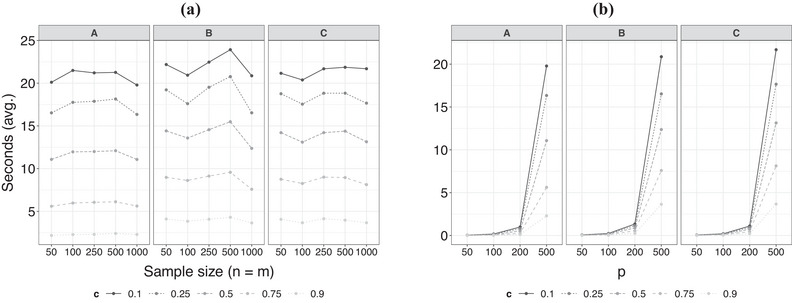
Average computational time required to estimate intervals of local maximum divergence for different sample sizes in (a) and grid resolution levels in (b).

**Computational complexity**. We also study the numerical efficiency of the proposed method using the Monte Carlo simulations study.^2^ In Figure 4(a), we depict the average time (in seconds) required to execute Algorithm 1 for different sample sizes and fix p=500 (the largest value in the Monte Carlo simulation) under the three scenarios, while and in panel (b), we fix the sample size at its largest value n=m=1000 and consider different grid resolutions levels p. As can be seen in both panels, small values of c (short intervals) involve more computation work since Algorithm 1 proceeds in an exhaustive search for the maxima. However, even in the case of c=0.1, m=n=1000, and p=500 (the shortest interval in the experiments and the largest data sets), it takes no more than 20 s (on average) to estimate the interval of local maximum divergence. In addition, the computational time remains constant on average as the sample size increases for fixed p and c 3; meanwhile, for fixed n, m, and c, the computational time increases exponentially in p. Redefining Step 2 in Algorithm 1 in order to search for a local maxima in a more efficient way will be part of the future research directions in order to tackle domain selection problems in the context of extremely large values of p.

## 4 Monitoring Electrocardiogram Signals

The ECG signal is the visual representation of the heart electrical activity as a function of time. Learning which part of the signal spectrum is more relevant to diagnose a cardiac disease, is of fundamental importance in order to increase the probability of survival during a cardiac episode. In this section, we illustrate the relevance of the proposed method in the analysis of ECG data.

**Data and goals**. The ECG data set (Olszewski 2001) is available in the UEA & UCR repository. It consists of 200 signals sampled over a grid of 96 equally spaced instances.^4^ Each observation represents the cardiac electrical activity recorded during one heartbeat and there are two groups of signals: 133 normal heartbeats and 67 myocardial infarction signals. The inferential and predictive tasks relevant in this section are: (i) Learn about $A^{*}\left(c\right)$ from data and quantify the uncertainty around the estimation of such interval—we use the bootstrap strategy described in Subsection 2.3. (ii) Although learning about $A^{*}\left(c\right)$ is, in principle, unrelated to classification, the ECG data are a popular benchmark for new classifiers; it may be sensible to ask whether the accuracy of DA^5^ can be improved by focusing on ${\hat{A}}^{*}\left(c\right)$ rather than treating the entire time horizon T equally.

**FIGURE 5:**
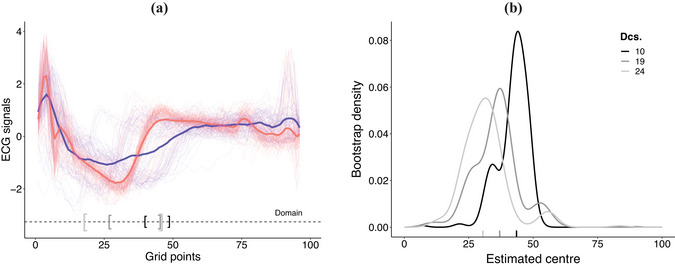
(a) ECG signals (lighted colored curves) alongside the estimated mean functions—solid curves corresponding to healthy ( **—** ) and myocardial infarction ( **—** ) signals. Selected domain for interval lengths 10,19, and 24 dcs displayed with black ”[ ‐ ]”, gray ”[ ‐ ]”, and light gray ”[ ‐ ]” brackets, respectively. (b) Bootstrap densities for the interval center (the median of each empirical distribution is reported on the horizontal axis) corresponding to intervals lengths 10,19, and 24 dcs.

**Implementation and results**. To learn about $A^{*}\left(c\right)$, we consider c∈{0.1,0.2,0.25} that corresponds to intervals of 10, 19, and 24 dcs, respectively. In Figure 5(a), we display the ECG raw signals along with the corresponding estimates ${\hat{A}}^{*}\left(c\right)$ using brackets. The selected domains correspond to grid points between 20 and 55. All in all, for small values of c, the analysis suggests that while normal heartbeats and myocardial infarction signals have similar “peaks” at the beginning of the sample period (i.e., they have similar Q waves, in ECG signal analysis terminology), immediately right after that period (i.e., over their ST segments) they greatly differ. To quantify the variability of our estimates, we replicate the estimation of ${\hat{A}}^{*}\left(c\right)$ using B=1000 bootstrap samples. Figure 5(b) displays the empirical density of the center of ${\hat{A}}^{*}\left(c\right)$ (we denote this distribution as ${\hat{F}}_{B}\left(t^{*}\right)$ in § 2.3) obtained via the bootstrap samples and suggests low variability in our estimator ${\hat{A}}^{*}\left(c\right)$ for c∈{0.1,0.2,0.25}.

**FIGURE 6:**
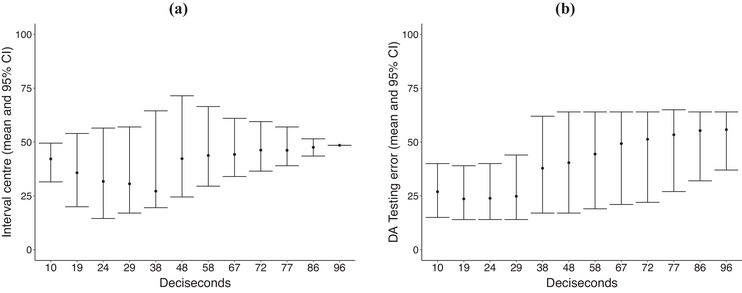
(a) Bootstrap interval center and 95% bootstrap CI throughout all the interval lengths considered. (b) Classification error using a discriminant model for different selected domains.

To assess the classification performance of DA over different slices on the domain, we consider c∈{0.1,0.2,0.25,0.3,0.4,0.5,0.6,0.7,0.75,0.8,0.9,1.0} that corresponds to intervals of 10,19,24,29,38,48,58,67,72,77,86, and 96 dcs, respectively; and randomly split the data into training–testing samples in a 50%–50% fashion. For each value of c, we learn ${\hat{A}}^{*}\left(c\right)$ and the corresponding parameters of the discriminant function with train data; while test data are used to estimate the misclassification error rate $\hat{\text{err}}\left(c\right)$. To assess the estimation variability on ${\hat{A}}^{*}\left(c\right)$ and $\hat{\text{err}}\left(c\right)$, we consider B=1000 bootstrap samples with train and test data, respectively. In Figure 6(a), we show the estimated centers of ${\hat{A}}^{*}\left(c\right)$ (black dots in the vertical box plots correspond to ${\hat{t}}_{c}^{*}$) and the corresponding 95% bootstrap confidence interval for such centers as a function of c (in a deciseconds scale). In Figure 6(b), we display the estimated $\hat{\text{err}}\left(c\right)$ (black dots in the vertical box plots) and its corresponding 95% bootstrap confidence interval as a function of c (in a deciseconds scale). As can be seen from Figure 5(b), the discrimination power of DA is significantly larger if we consider a small interval of local maximum divergence on the ECG data—say c∈{0.1,0.2,0.25,0.3}—rather than considering the full domain corresponding to ECG data.

## 5 Discussion and Closing Remarks

From a methodological outlook, the main goal of this article entails the combination of Kullback–Leibler divergence and GPs to develop a superfast and easy to implement algorithm for *domain selection*. We define a local KL divergence, introduce its fundamental properties, and prove the existence of an interval of local maximum divergence $A^{*}\left(c\right)$ under suitable conditions on the mean and variance functions. In addition, we also propose an estimator for $A^{*}\left(c\right)$, devise a nonparametric approach to assess the estimation uncertainty, and also discuss relevant variants and extensions.

Through a Monte Carlo simulation study, we numerically assess the consistency of our estimator and demonstrate that even for large n and p (i.e., n=m=1000 and p=500, which corresponds to high‐dimensional and large data sets), Algorithm 1 is very efficient. Learning about intervals of local maximum divergence in the context ECG data contributes to improve diagnostic tools, as we demonstrate in the analysis of ECG data. In addition, we also explore how the discrimination power of DA (healthy vs. disease heartbeats) can be improved by making emphasis on a small interval of ECG data rather than using the full domain.

Although the proposed method can be used for domain selection with GP, there are natural opportunities for further analysis: (i) In this paper, consistency is illustrated with a Monte Carlo simulation. Further theoretical developments will be conducted in order to establish general conditions in the model in order to ensure that ${\hat{A}}^{*}\left(c\right)$ is a consistent estimator for $A^{*}\left(c\right)$. (ii) To define local KL divergences for GP, we rely on a discretized version of X(t) and Y(t); therefore, a natural point to address is to extend Equations (2) and (3) to the nondiscretized case; studying also the impact on the numerical complexity of adapting Algorithm 1 to such context. (iii) Another important avenue for future research is the extension of the proposed domain selection method to address non‐GP data, perhaps using alternative metrics to assess differences between non‐GPs. (iv) In the framework of curve classification, it is important to balance predictive classification accuracy against the number of covariates that, in the case of curves, corresponds to the length of the interval. Therefore, a natural follow‐up within the remit of this paper is the analysis of domain selection as a shrinkage method for curve classification. (v) We also propose to study alternative ways to introduce Bayesian tools for domain selection with GP as we discuss in the paragraph *Sampling Designs* in § 2.2. (vi) Finally, from a computational view point, Step 2 in Algorithm 1 needs to be reformulated for extremely high‐dimensional data since its computational complexity grows exponentially in p as we mention in Section 2 and illustrate numerically in Section 3.

## Authors Contributions

Authors contributed equally to this work.

## Ethics Approval

Authors have no affiliations with or involvement in any organization or entity with any financial interest or nonfinancial interest in the subject matter or materials discussed in this manuscript.

## Consent for Publication

Authors give consent for publication.

## Availability of Data and Code

Data are available on UEA & UCR and source code to reproduce the results is included as a supplementary file in the submission.

## Conflicts of Interest

None of the authors has a conflict of interest.

### Open Research Badges

This article has earned an Open Data badge for making publicly available the digitally‐shareable data necessary to reproduce the reported results. The data is available in the Supporting Information section.

This article has earned an open data badge “**Reproducible Research**” for making publicly available the code necessary to reproduce the reported results. The results reported in this article could fully be reproduced.

## Supporting information

Supporting Information

## Data Availability

The data that support the findings of this study are available in the Supporting Information of this article.

## Footnotes

1 Notice that $r_{c}$ is a nuisance parameter that only depends on c.

2 The experiment was executed in a computer with 36 cores and 1.5TB per node, paralleling per Monte Carlo iteration, sample size, grid resolution, and scenario.

3 Notice that the time required to compute means and variances does not increase in a sensitive way as n and m increase. Since mean and variances are estimated only once in Algorithm 1, then increasing n and m has approximately zero impact in the computational time required to estimate local divergences and the corresponding interval of local maximum divergence.

4 A healthy heart runs on a typical rate of 70–75 beats per minute and take about 0.8 s to complete the cardiac cycle.

5 DA is the optimal classifier for Gaussian data; nevertheless, other classifiers can be considered as well. The analysis of other classification methods reaches out of the scope of this paper.

## Appendix

We state the auxiliary result that will be used to prove part (b) in Proposition 2.1.
Let X and Y be two random vectors in $R^{p}$ with density functions $f_{X}\left(x\right)$ and $f_{Y}\left(y\right)$. For all partitions $X\equiv (X_{1},X_{2})$ and $Y\equiv (Y_{1},Y_{2})$, where $X_{1}$ and $Y_{1}$ are random vectors in $R^{q}$ with 1≤q<p, then it holds that $\text{KL}(X||Y)\geq \text{KL}(X_{1}\left|\right|Y_{1})$.

Proof of Lemma A Consider the factorization: $f_{X}\left(x\right)=f_{X_{1}}\left(x_{1}\right)f_{X_{2}|X_{1}}\left(x_{2}|x_{1}\right)$ and $f_{Y}\left(y\right)=f_{Y_{1}}\left(y_{1}\right)f_{Y_{2}|Y_{1}}\left(y_{2}|y_{1}\right)$, where $f_{X_{2}|X_{1}}\left(x_{2}|x_{1}\right)$ and $f_{Y_{2}|Y_{1}}\left(y_{2}|y_{1}\right)$ denote the corresponding densities of conditional random vectors $X_{2}|X_{1}$ and $Y_{2}|Y_{1}$, respectively, then:

$$\begin{array}{ccc} \text{KL}(X||Y) & = & \;\int \int \;f_{X_{1}}\left(u\right)f_{X_{2}|X_{1}}\left(v|u\right)\ln\left(\frac{f_{X_{1}}\left(u\right)f_{X_{2}|X_{1}}\left(v|u\right)}{f_{Y_{1}}\left(u\right)f_{Y_{2}|Y_{1}}\left(v|u\right)}\right)dudv,\\ & = & \int f_{X_{1}}\left(u\right)\ln\left(\frac{f_{X_{1}}\left(u\right)}{f_{Y_{1}}\left(u\right)}\right)du\\ & & +\int f_{X_{1}}\left(u\right)\int f_{X_{2}|X_{1}}\left(v|u\right)\ln\left(\frac{f_{X_{2}|X_{1}}\left(v|u\right)}{f_{Y_{2}|Y_{1}}\left(v|u\right)}\right)dvdu,\\ & = & \text{KL}(X_{1}\left|\right|Y_{1})+E_{X_{1}}\{\text{KL}(X_{2}|X_{1}\left|\right|Y_{2}|Y_{1})\}, \end{array}$$

and the inequality $\text{KL}(X||Y)\geq \text{KL}(X_{1}\left|\right|Y_{2})$ holds since $\text{KL}(X_{2}|X_{1}||Y_{2}|Y_{1})\geq 0$ almost surely.□

Proof of Proposition 2.1 To prove the upper bound in Property (b), consider $\Gamma \equiv \Sigma_{T,Y}^{-1/2}\Sigma_{T,X}\Sigma_{T,Y}^{-1/2}$, where $\Sigma_{T,X}$ and $\Sigma_{T,Y}$ are symmetric PD matrices because $\sigma_{X}\left(s,t\right)$ and $\sigma_{Y}\left(s,t\right)$ are PD functions. Notice that the p=|T| eigenvalues $\infty >\gamma_{1}\geq \cdots \geq \gamma_{p}>0$ corresponding to matrix Γ are nonnegative, and $\frac{\text{det}(\Sigma_{T,X})}{\text{det}(\Sigma_{T,Y})}=\text{det}\left(\Gamma \right)$ and $\text{tr}\left(\Sigma_{T,X}\Sigma_{T,Y}^{-1}\right)=\text{tr}\left(\Gamma \right)$. Therefore, under continuity in $\mu_{X}\left(t\right)$ and $\mu_{Y}\left(t\right)$ over the compact set T (i.e., $∥\Delta_{T}{∥}_{2}<\infty$):

$$2{\text{KL}}_{T}\left(X||Y\right)=\sum_{i=1}^{p}\{\gamma_{i}-\ln\left(\gamma_{i}\right)\}-p+\Delta_{T}^{T}\Sigma_{T,Y}^{-1}\Delta_{T}<\infty ,$$

since 1≤x−ln(x)<∞, for all 0<x<∞. In addition, ${\text{KL}}_{T}\left(X||Y\right)=0$ implies that $\gamma_{1}=\cdots =\gamma_{p}=1$ and $\Delta_{T}=0$. Monotonicity is a consequence of Lemma A: For any nonempty subset $A^{\prime }\subset A$, the random vectors $X_{A}$ and $Y_{A}$ can be partitioned as $X_{A}=\left(X_{A^{\prime }},X_{A^{\prime \prime }}\right)$ and $Y_{A}=\left(Y_{A\prime },Y_{A^{\prime \prime }}\right)$, where $A^{\prime \prime }=A\cap {\left(A^{\prime }\right)}^{C}$. Then, the inequality ${\text{KL}}_{A}\left(X||Y\right)\geq {\text{KL}}_{A^{\prime }}\left(X||Y\right)$ follows from Lemma A:

$${\text{KL}}_{A}(X||Y)=\text{KL}(X_{A}\left|\right|Y_{A})\geq \text{KL}(X_{A^{\prime }}\left|\right|Y_{A^{\prime }})={\text{KL}}_{A^{\prime }}\left(X||Y\right).$$

Property (a) follows from general properties of KL divergence. In addition, ${\text{KL}}_{T}\left(X||Y\right)=0$ implies that $\gamma_{1}=\cdots =\gamma_{p}=1$ and $\Delta_{T}=0$, which completes the proof.□
