# Supplementary material for: Domain Selection for Gaussian Process Data: An Application to Electrocardiogram Signals
Source: Biom J. 2024 Nov 28;66(8):e70018. doi: 10.1002/bimj.70018 (PMC11604031; doi:10.1002/bimj.70018)
Supplement: Supplementary file 1 — Supporting Information [file BIMJ-66-e70018-s001.zip › KL4GP-Reproducibility/README FILE.rtf]

################################################ README FILE ################################################This readme file is a guide to reproduce the results of the manuscript: “Domain Selection for Gaussian Process Data: An application to electrocardiogram signals”.Before running any code is important to i) install the required packages and ii) check the path where files and figures are saved.The zip file has 4 folders.	¥DATA: Contains the data split in Train and Test for the Real Data Experiment in Section 4: “Monitoring electrocardiogram signals”. It also contains *.Rdata files needed as input for the figures.	¥SCTIPTS: Contains the R files to reproduce the experiments of the paper as well as any example figures. Note: It is not necessary to run this experiment to reproduce Fig. 3 to Fig 6, results are provided in folder ‘Data’ in Rdata format.	¥FIGURES: Contains the Figures generated by the respective scripts	¥RESULTS: Contains the *.Rdata files as results of the experiments and input of the figures.Description of files inside SCRIPTSFILE id	FILE NAME	DESCRIPTION	
1	Experiment_Section_3_processing_times.R	Code to reproduce Results of Experiment in Section 3. Note: It is not necessary to run this experiment to reproduce Fig. 4. Results are provided in folder ‘Data’. ‘L = 500’ are the MC replicates (do not need to be modified).	
2	Experiment_Section_3_Scenario_A.R	Code to reproduce Results of Experiment in Section 3.  Note: It is not necessary to run this experiment to reproduce Fig. 3. Results are provided in folder ‘Data’. ‘eigen.tol = 1e-3’ represents the threshold for the eigenvalue in the covariance matrix inversion. Following experiment description in section 3 of the paper, sample size ’n’ takes the values: 50, 100, 250, 500 and 1000. So n = 100 in line 15 must be replaced by  50, 250, 500 and 1000 iteratively to get the full results. Moreover, the grid resolution ‘p’ takes the value: 50, 100, 200 and 500. So p = 50 in line 16 must be replaced by 100, 200 and 500 iteratively to get the full results.	
3	Experiment_Section_3_Scenario_B.R	Code to reproduce Results of Experiment in Section 3.  Note: It is not necessary to run this experiment to reproduce Fig. 3. Results are provided in folder ‘Data’. ‘eigen.tol = 1e-3’ represents the threshold for the eigenvalue in the covariance matrix inversion. Following experiment description in section 3 of the paper, sample size ’n’ takes the values: 50, 100, 250, 500 and 1000. So n = 100 in line 15 must be replaced by  50, 250, 500 and 1000 iteratively to get the full results. Moreover, the grid resolution ‘p’ takes the value: 50, 100, 200 and 500. So p = 50 in line 16 must be replaced by 100, 200 and 500 iteratively to get the full results.	
4	Experiment_Section_3_Scenario_C.R	Code to reproduce Results of Experiment in Section 3.  Note: It is not necessary to run this experiment to reproduce Fig. 4. Results are provided in folder ‘Data’. ‘eigen.tol = 1e-3’ represents the threshold for the eigenvalue in the covariance matrix inversion. Following experiment description in section 3 of the paper, sample size ’n’ takes the values: 50, 100, 250, 500 and 1000. So n = 100 in line 15 must be replaced by  50, 250, 500 and 1000 iteratively to get the full results. Moreover, the grid resolution ‘p’ takes the value: 50, 100, 200 and 500. So p = 50 in line 16 must be replaced by 100, 200 and 500 iteratively to get the full results.	
5	Experiment_Section_4.R	Code to reproduce Results of Experiment in Section 4. ‘B = 1000’ represents the bootstrap samples and ‘eigen.tol = 0.001’ represents the threshold for the eigenvalue in the covariance matrix inversion.	
6	Fig3_a_b_c.R	Code to reproduce Boxplots to display results of Monte Carlo experiment in Section 3	
7	Fig4_a_b.R	Code to reproduce plots to display computational times results of Monte Carlo experiment in Section 3	
8	Fig5_a_b.R	Code to reproduce domain selection results of real data experiment in Section 4	
9	Fig6_a_b.R	Code to reproduce classification results under domain selection in real data experiment in Section 4	
10	Figure1_a_b.R	Code to reproduce example Figure 1 (a and b).	
11	Figure2_a_d.R	Code to reproduce One-Shot experiment under Scenario A, in Figure 2 (subfigure a and d)	
12	Figure2_b_e.R	Code to reproduce One-Shot experiment under Scenario B, in Figure 2 (subfigure b and e)	
13	Figure2_c_f.R	Code to reproduce One-Shot experiment under Scenario C, in Figure 2 (subfigure c and f)	
14	Compiling_Data_Results.R	Compiles the results of Experiment in Section 3. The output is 'AJD.Rdata' which is the input for the sctipt (File id #6)	
15	master.R	Run all the scripts	
> sessionInfo()R version 4.2.1 (2022-06-23)Platform: x86_64-apple-darwin17.0 (64-bit)Running under: macOS 14.5Matrix products: defaultLAPACK: /Library/Frameworks/R.framework/Versions/4.2/Resources/lib/libRlapack.dyliblocale:[1] en_US.UTF-8/en_US.UTF-8/en_US.UTF-8/C/en_US.UTF-8/en_US.UTF-8attached base packages:[1] splines   stats     graphics  grDevices utils     datasets  methods   base     other attached packages: [1] pracma_2.4.4      ASSA_2.0          forcats_0.5.2     stringr_1.4.1     [5] dplyr_1.0.10      purrr_0.3.5       readr_2.1.3       tidyr_1.2.1       [9] tibble_3.2.1      tidyverse_1.3.2   fda_6.1.4         deSolve_1.38     [13] fds_1.8           RCurl_1.98-1.13   rainbow_3.7       pcaPP_2.0-3      [17] data.table_1.14.2 MASS_7.3-57       ggplot2_3.4.4    loaded via a namespace (and not attached): [1] mclust_6.0.0        lubridate_1.8.0     mvtnorm_1.2-3       lattice_0.20-45     [5] assertthat_0.2.1    utf8_1.2.4          R6_2.5.1            cellranger_1.1.0    [9] backports_1.4.1     reprex_2.0.2        httr_1.4.4          pillar_1.9.0       [13] rlang_1.1.2         googlesheets4_1.0.1 readxl_1.4.1        rstudioapi_0.14    [17] Matrix_1.5-1        googledrive_2.0.0   munsell_0.5.0       broom_1.0.1        [21] compiler_4.2.1      modelr_0.1.9        pkgconfig_2.0.3     tidyselect_1.2.0   [25] hdrcde_3.4          fansi_1.0.5         crayon_1.5.2        tzdb_0.3.0         [29] dbplyr_2.2.1        withr_2.5.2         bitops_1.0-7        grid_4.2.1         [33] jsonlite_1.8.7      gtable_0.3.4        lifecycle_1.0.4     DBI_1.1.3          [37] magrittr_2.0.3      scales_1.2.1        KernSmooth_2.23-20  stringi_1.7.8      [41] cli_3.6.1           fs_1.6.3            xml2_1.3.3          ellipsis_0.3.2     [45] generics_0.1.3      vctrs_0.6.4         tools_4.2.1         glue_1.6.2         [49] hms_1.1.2           ks_1.14.1           colorspace_2.1-0    gargle_1.2.1       [53] cluster_2.1.3       rvest_1.0.3         haven_2.5.1      
