# Supplementary material for: Domain Selection for Gaussian Process Data: An Application to Electrocardiogram Signals
Source: Biom J. 2024 Nov 28;66(8):e70018. doi: 10.1002/bimj.70018 (PMC11604031; doi:10.1002/bimj.70018)
Supplement: Supplementary file 1 — Supporting Information [file BIMJ-66-e70018-s001.zip › KL4GP-Reproducibility/README-FILE.pdf]

#####################################
############ README FILE ############
#####################################

This readme file is a guide to reproduce the results of the manuscript: “Domain Selection for Gaussian Process Data: An application to electrocardiogram signals”.

Before running any code is important to i) install the required packages and ii) check the path where files and figures are saved.

The zip file has 4 folders.

- **DATA**: Contains the data split in Train and Test for the Real Data Experiment in Section 4: “Monitoring electrocardiogram signals”. It also contains *.Rdata files needed as input for the figures.
- SCTIPTS: Contains the R files to reproduce the experiments of the paper as well as any example figures. Note: It is not necessary to run this experiment to reproduce Fig. 3 to Fig 6, results are provided in folder ‘Data’ in Rdata format.
- FIGURES: Contains the Figures generated by the respective scripts
- RESULTS: Contains the *.Rdata files as results of the experiments and input of the figures.

Description of files inside SCRIPTS

| FILE id | FILE NAME | DESCRIPTION |
|---|---|---|
| 1 | Experiment_Section_3_processing_times.R | Code to reproduce Results of Experiment in Section 3. Note: It is not necessary to run this experiment to reproduce Fig. 4. Results are provided in folder ‘Data’. ‘L = 500’ are the MC replicates (do not need to be modified). |
| 2 | Experiment_Section_3_Scenario_A.R | Code to reproduce Results of Experiment in Section 3. Note: It is not necessary to run this experiment to reproduce Fig. 3. Results are provided in folder ‘Data’. ‘eigen.tol = 1e-3’ represents the threshold for the eigenvalue in the covariance matrix inversion. Following experiment description in section 3 of the paper, sample size ’n’ takes the values: 50, 100, 250, 500 and 1000. So n = 100 in line 15 must be replaced by 50, 250, 500 and 1000 iteratively to get the full results. Moreover, the grid resolution ‘p’ takes the value: 50, 100, 200 and 500. So p = 50 in line 16 must be replaced by 100, 200 and 500 iteratively to get the full results. |
| 3 | Experiment_Section_3_Scenario_B.R | Code to reproduce Results of Experiment in Section 3. Note: It is not necessary to run this |

| | | |
|---|---|---|
| | | experiment to reproduce Fig. 3. Results are provided in folder ‘Data’. ‘eigen.tol = 1e-3’ represents the threshold for the eigenvalue in the covariance matrix inversion. Following experiment description in section 3 of the paper, sample size ’n’ takes the values: 50, 100, 250, 500 and 1000. So n = 100 in line 15 must be replaced by 50, 250, 500 and 1000 iteratively to get the full results. Moreover, the grid resolution ‘p’ takes the value: 50, 100, 200 and 500. So p = 50 in line 16 must be replaced by 100, 200 and 500 iteratively to get the full results. |
| 4 | Experiment_Section_3_Scenario_C.R | Code to reproduce Results of Experiment in Section 3. Note: It is not necessary to run this experiment to reproduce Fig. 4. Results are provided in folder ‘Data’. ‘eigen.tol = 1e-3’ represents the threshold for the eigenvalue in the covariance matrix inversion. Following experiment description in section 3 of the paper, sample size ’n’ takes the values: 50, 100, 250, 500 and 1000. So n = 100 in line 15 must be replaced by 50, 250, 500 and 1000 iteratively to get the full results. Moreover, the grid resolution ‘p’ takes the value: 50, 100, 200 and 500. So p = 50 in line 16 must be replaced by 100, 200 and 500 iteratively to get the full results. |
| 5 | Experiment_Section_4.R | Code to reproduce Results of Experiment in Section 4. ‘B = 1000’ represents the bootstrap samples and ‘eigen.tol = 0.001’ represents the threshold for the eigenvalue in the covariance matrix inversion. |
| 6 | Fig3_a_b_c.R | Code to reproduce Boxplots to display results of Monte Carlo experiment in Section 3 |
| 7 | Fig4_a_b.R | Code to reproduce plots to display computational times results of Monte Carlo experiment in Section 3 |
| 8 | Fig5_a_b.R | Code to reproduce domain selection results of real data experiment in Section 4 |
| 9 | Fig6_a_b.R | Code to reproduce classification results under domain selection in real data experiment in Section 4 |
| 10 | Figure1_a_b.R | Code to reproduce example Figure 1 (a and b). |
| 11 | Figure2_a_d.R | Code to reproduce One-Shot experiment under Scenario A, in Figure 2 (subfigure a and d) |
| 12 | Figure2_b_e.R | Code to reproduce One-Shot experiment under Scenario B, in Figure 2 (subfigure b and e) |
| 13 | Figure2_c_f.R | Code to reproduce One-Shot experiment under |

| | | |
|---|---|---|
| | | Scenario C, in Figure 2 (subfigure c and f) |
| 14 | Compiling_Data_Results.R | Compiles the results of Experiment in Section 3. The output is 'AJD.Rdata' which is the input for the sctipt (File id #6) |
| 15 | master.R | Run all the scripts |

```
> sessionInfo()
R version 4.2.1 (2022-06-23)
Platform: x86_64-apple-darwin17.0 (64-bit)
Running under: macOS 14.5

Matrix products: default
LAPACK:
/Library/Frameworks/R.framework/Versions/4.2/Resources/lib/libRlapack.dylib

locale:
[1] en_US.UTF-8/en_US.UTF-8/en_US.UTF-8/C/en_US.UTF-8/en_US.UTF-8

attached base packages:
[1] splines   stats     graphics  grDevices utils     datasets  methods   base

other attached packages:
 [1] pracma_2.4.4      ASSA_2.0          forcats_0.5.2     stringr_1.4.1
 [5] dplyr_1.0.10      purrr_0.3.5       readr_2.1.3       tidyr_1.2.1
 [9] tibble_3.2.1      tidyverse_1.3.2   fda_6.1.4         deSolve_1.38
[13] fds_1.8           RCurl_1.98-1.13   rainbow_3.7       pcaPP_2.0-3
[17] data.table_1.14.2 MASS_7.3-57       ggplot2_3.4.4

loaded via a namespace (and not attached):
 [1] mclust_6.0.0        lubridate_1.8.0     mvtnorm_1.2-3       lattice_0.20-45
 [5] assertthat_0.2.1    utf8_1.2.4          R6_2.5.1            cellranger_1.1.0
 [9] backports_1.4.1     reprex_2.0.2        httr_1.4.4          pillar_1.9.0
[13] rlang_1.1.2         googlesheets4_1.0.1 readxl_1.4.1        rstudioapi_0.14
[17] Matrix_1.5-1        googledrive_2.0.0   munsell_0.5.0       broom_1.0.1
[21] compiler_4.2.1      modelr_0.1.9        pkgconfig_2.0.3     tidyselect_1.2.0
[25] hdrcde_3.4          fansi_1.0.5         crayon_1.5.2        tzdb_0.3.0
[29] dbplyr_2.2.1        withr_2.5.2         bitops_1.0-7        grid_4.2.1
[33] jsonlite_1.8.7      gtable_0.3.4        lifecycle_1.0.4     DBI_1.1.3
[37] magrittr_2.0.3      scales_1.2.1        KernSmooth_2.23-20  stringi_1.7.8
[41] cli_3.6.1           fs_1.6.3            xml2_1.3.3          ellipsis_0.3.2
[45] generics_0.1.3      vctrs_0.6.4         tools_4.2.1         glue_1.6.2
[49] hms_1.1.2           ks_1.14.1           colorspace_2.1-0    gargle_1.2.1
[53] cluster_2.1.3       rvest_1.0.3         haven_2.5.1
```
