# Supplementary figures and images for: Domain Selection for Gaussian Process Data: An Application to Electrocardiogram Signals
Source: Biom J. 2024 Nov 28;66(8):e70018. doi: 10.1002/bimj.70018 (PMC11604031; doi:10.1002/bimj.70018)

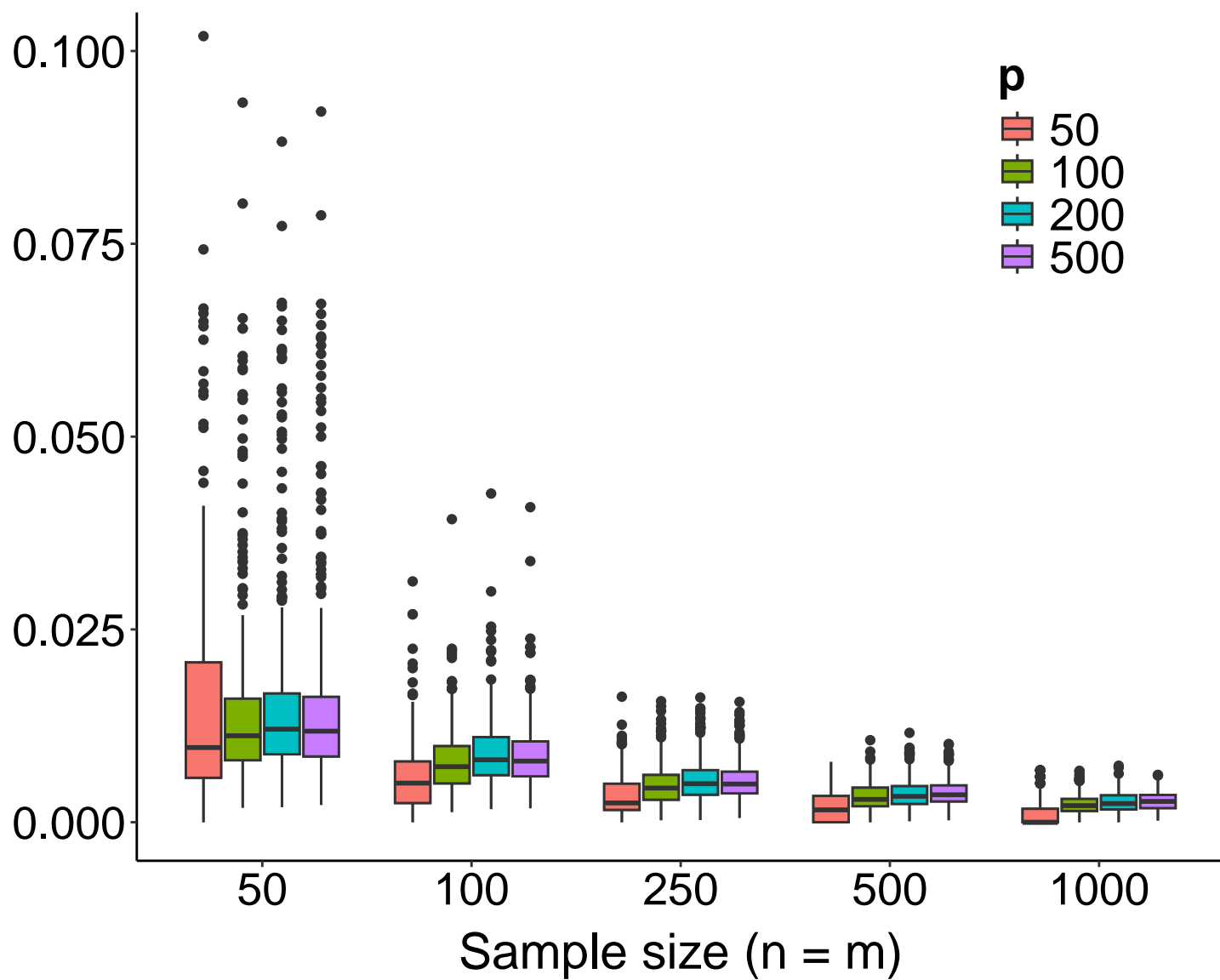

Supplement: Supplementary file 1 — Supporting Information [file BIMJ-66-e70018-s001.zip › KL4GP-Reproducibility/Figures/Fig3_a.pdf]

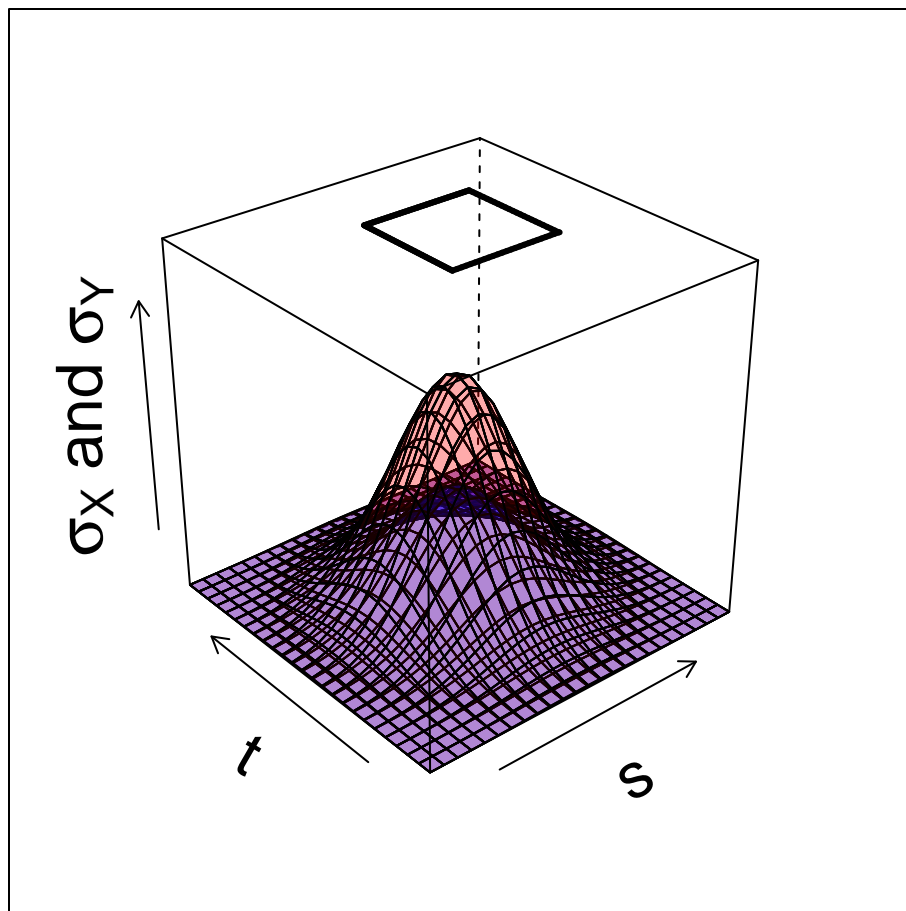

Supplement: Supplementary file 1 — Supporting Information [file BIMJ-66-e70018-s001.zip › KL4GP-Reproducibility/Figures/Fig1_b.pdf]

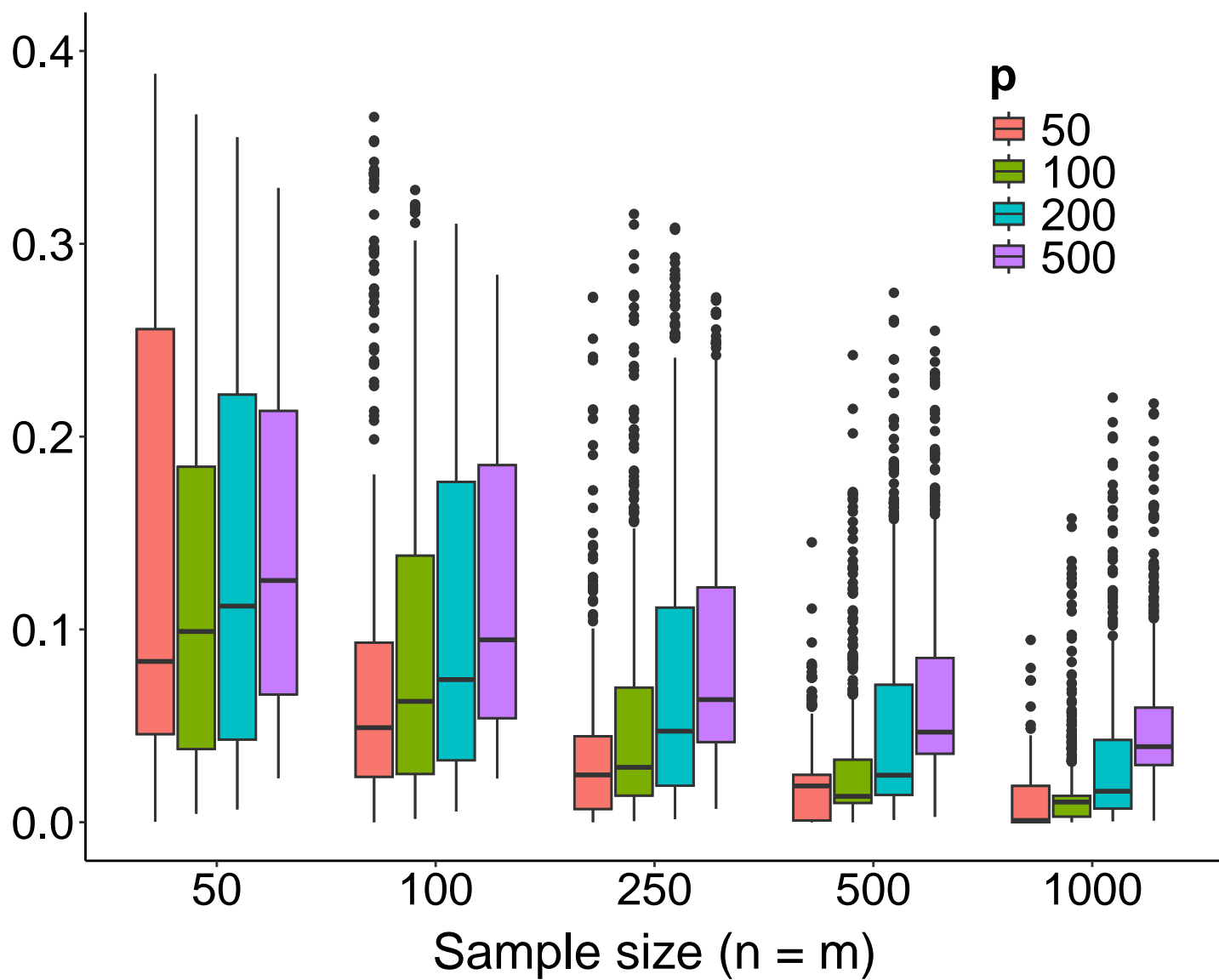

Supplement: Supplementary file 1 — Supporting Information [file BIMJ-66-e70018-s001.zip › KL4GP-Reproducibility/Figures/Fig3_b.pdf]

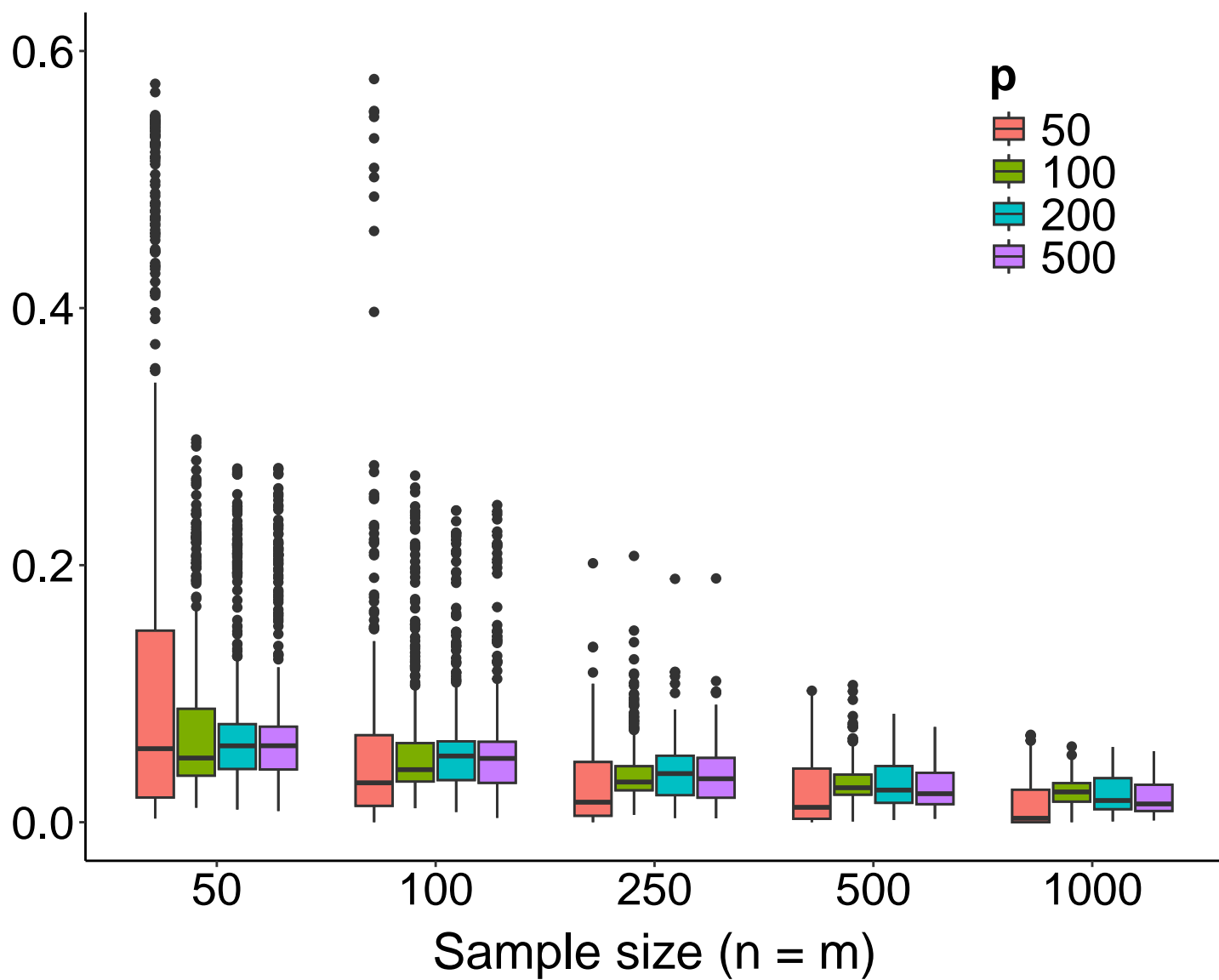

Supplement: Supplementary file 1 — Supporting Information [file BIMJ-66-e70018-s001.zip › KL4GP-Reproducibility/Figures/Fig3_c.pdf]

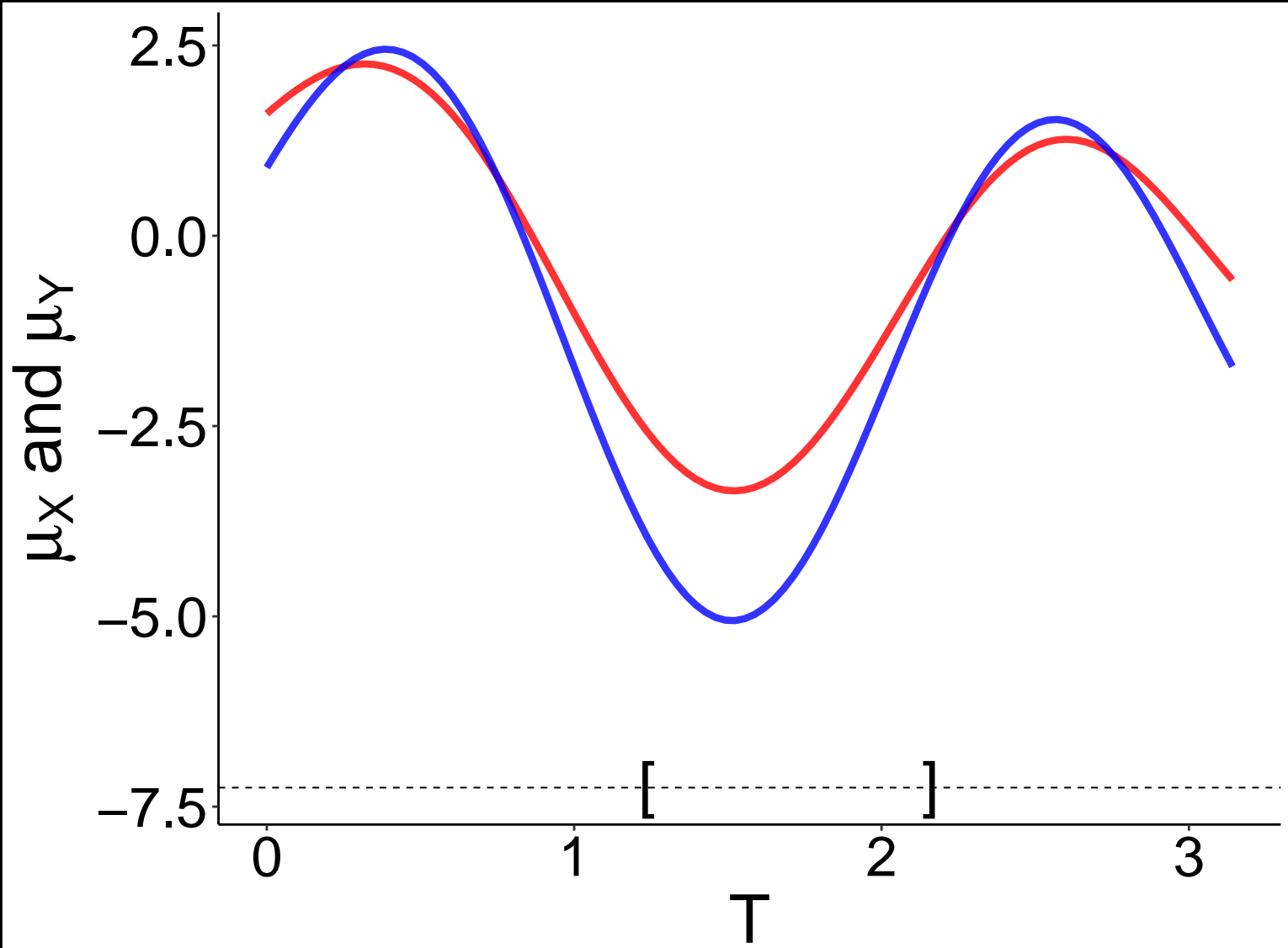

Supplement: Supplementary file 1 — Supporting Information [file BIMJ-66-e70018-s001.zip › KL4GP-Reproducibility/Figures/Fig1_a.pdf]

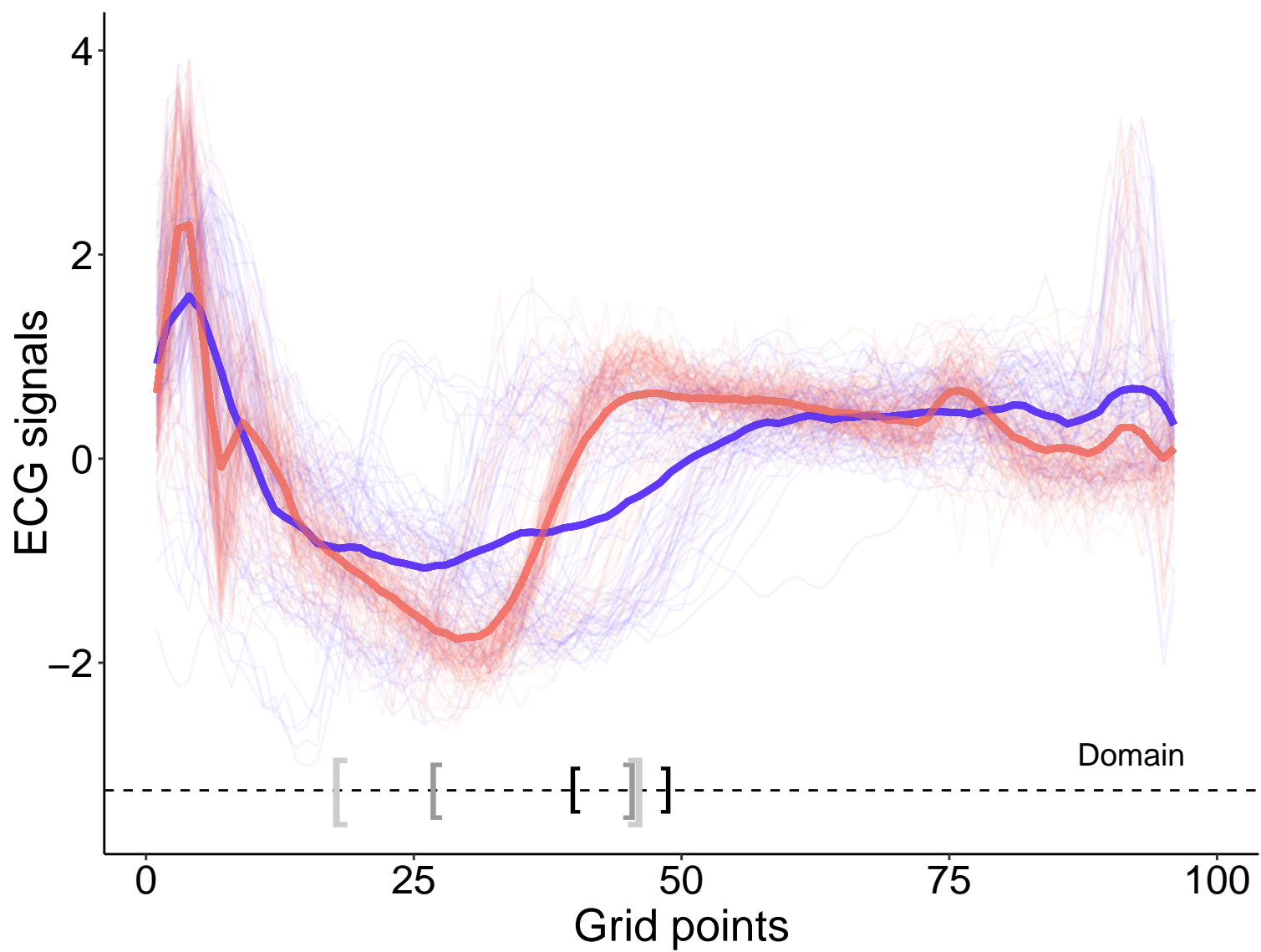

Supplement: Supplementary file 1 — Supporting Information [file BIMJ-66-e70018-s001.zip › KL4GP-Reproducibility/Figures/Fig5_a.pdf]

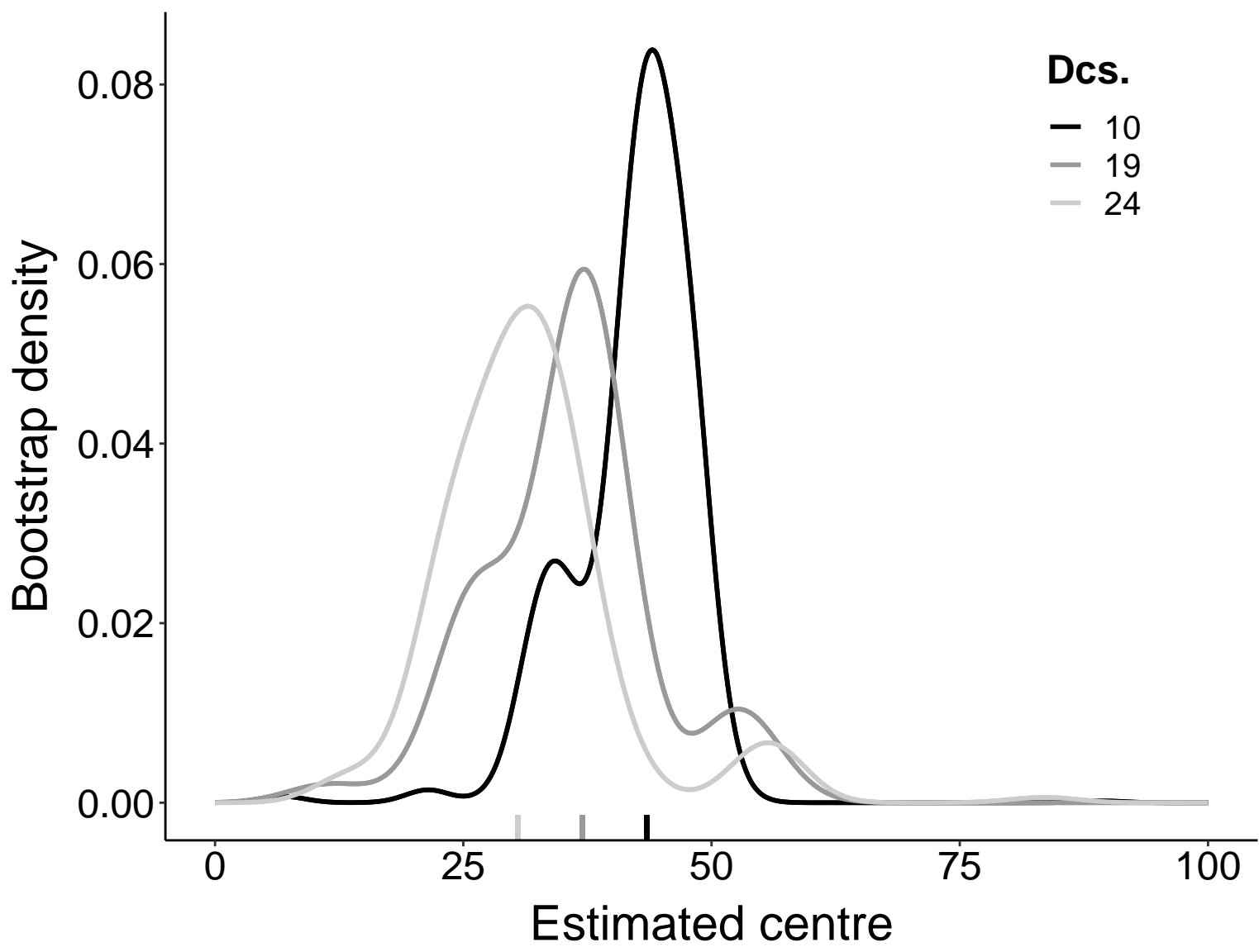

Supplement: Supplementary file 1 — Supporting Information [file BIMJ-66-e70018-s001.zip › KL4GP-Reproducibility/Figures/Fig5_b.pdf]

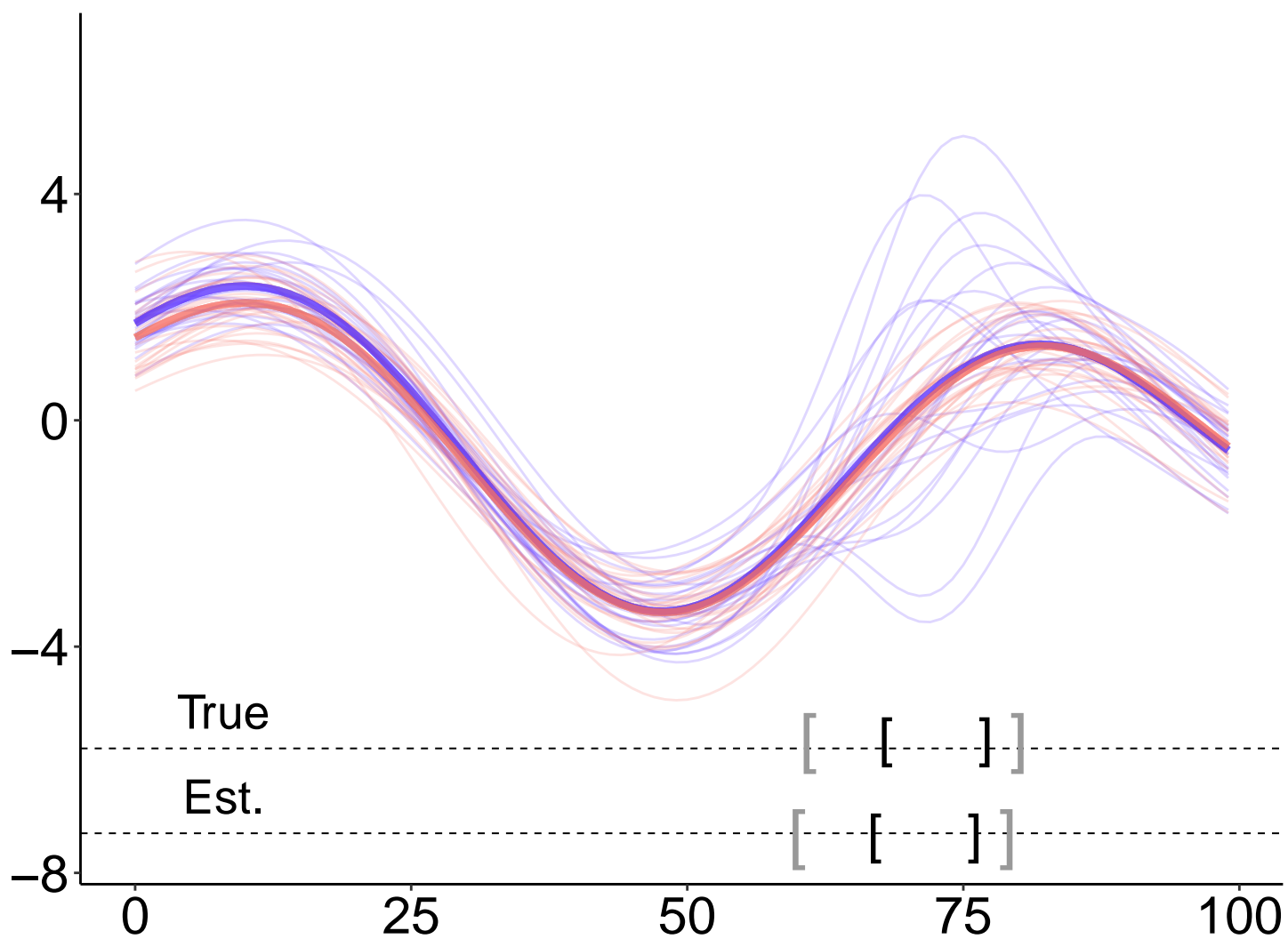

Supplement: Supplementary file 1 — Supporting Information [file BIMJ-66-e70018-s001.zip › KL4GP-Reproducibility/Figures/Fig2_b.pdf]

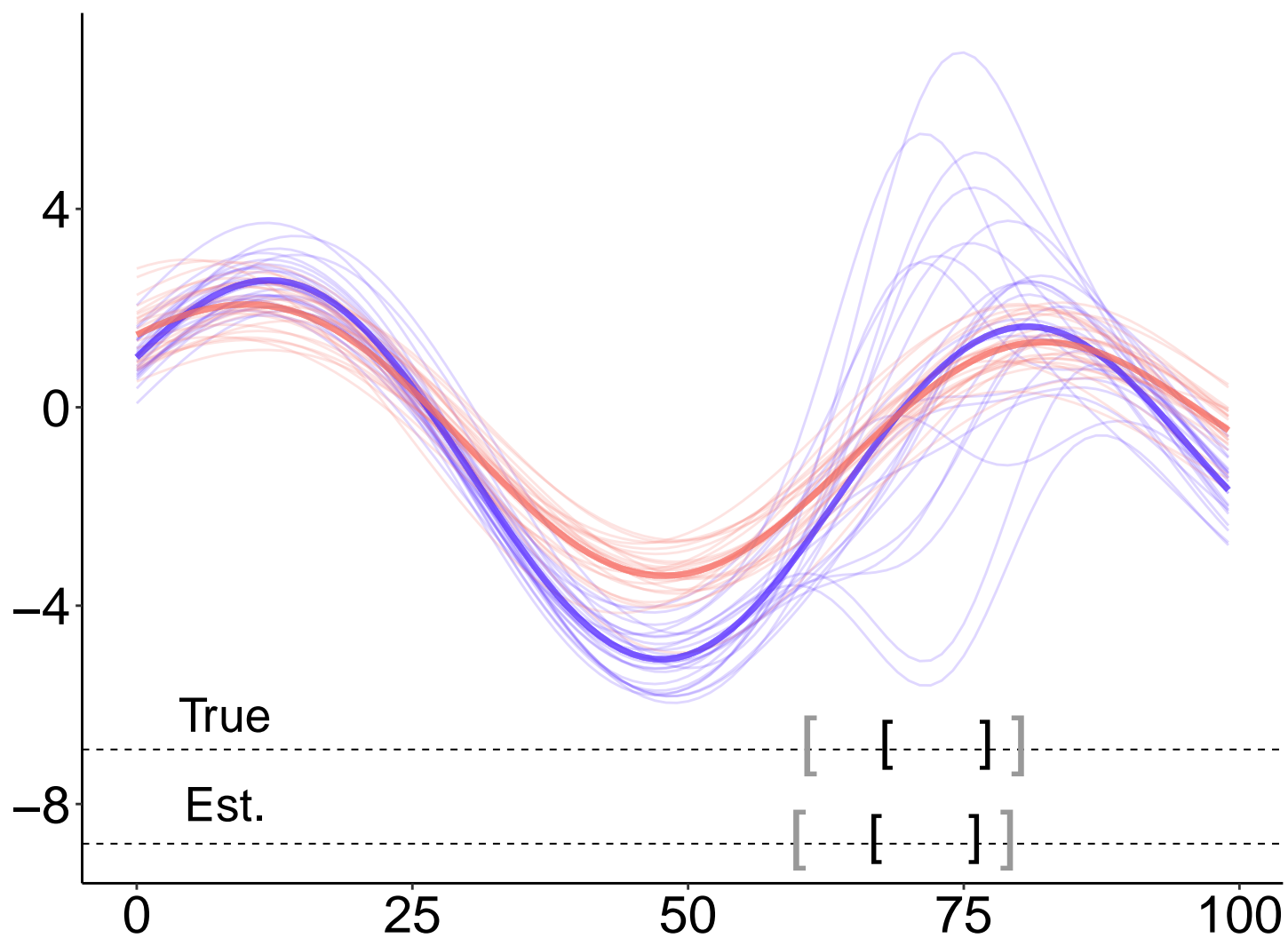

Supplement: Supplementary file 1 — Supporting Information [file BIMJ-66-e70018-s001.zip › KL4GP-Reproducibility/Figures/Fig2_c.pdf]

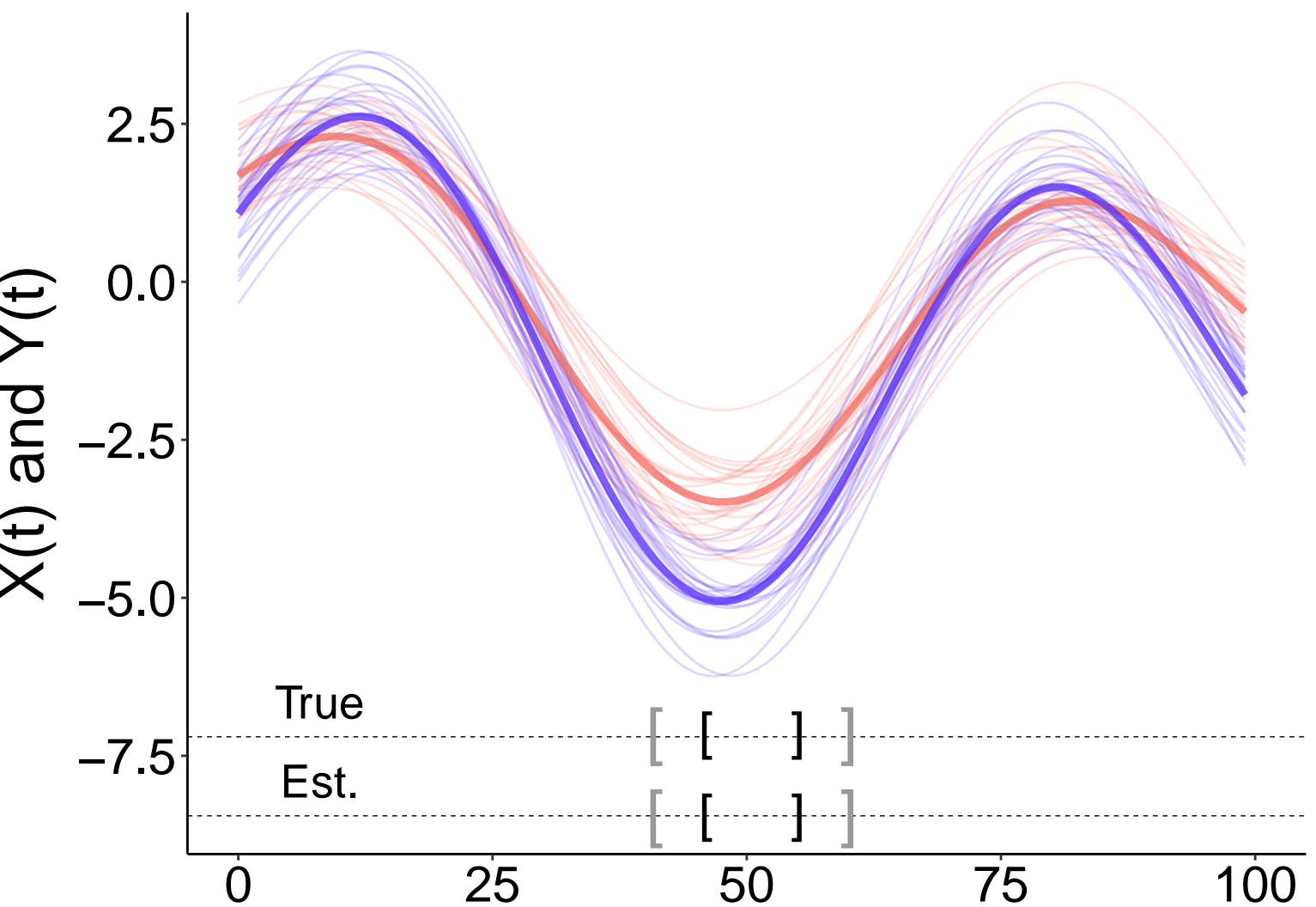

Supplement: Supplementary file 1 — Supporting Information [file BIMJ-66-e70018-s001.zip › KL4GP-Reproducibility/Figures/Fig2_a.pdf]

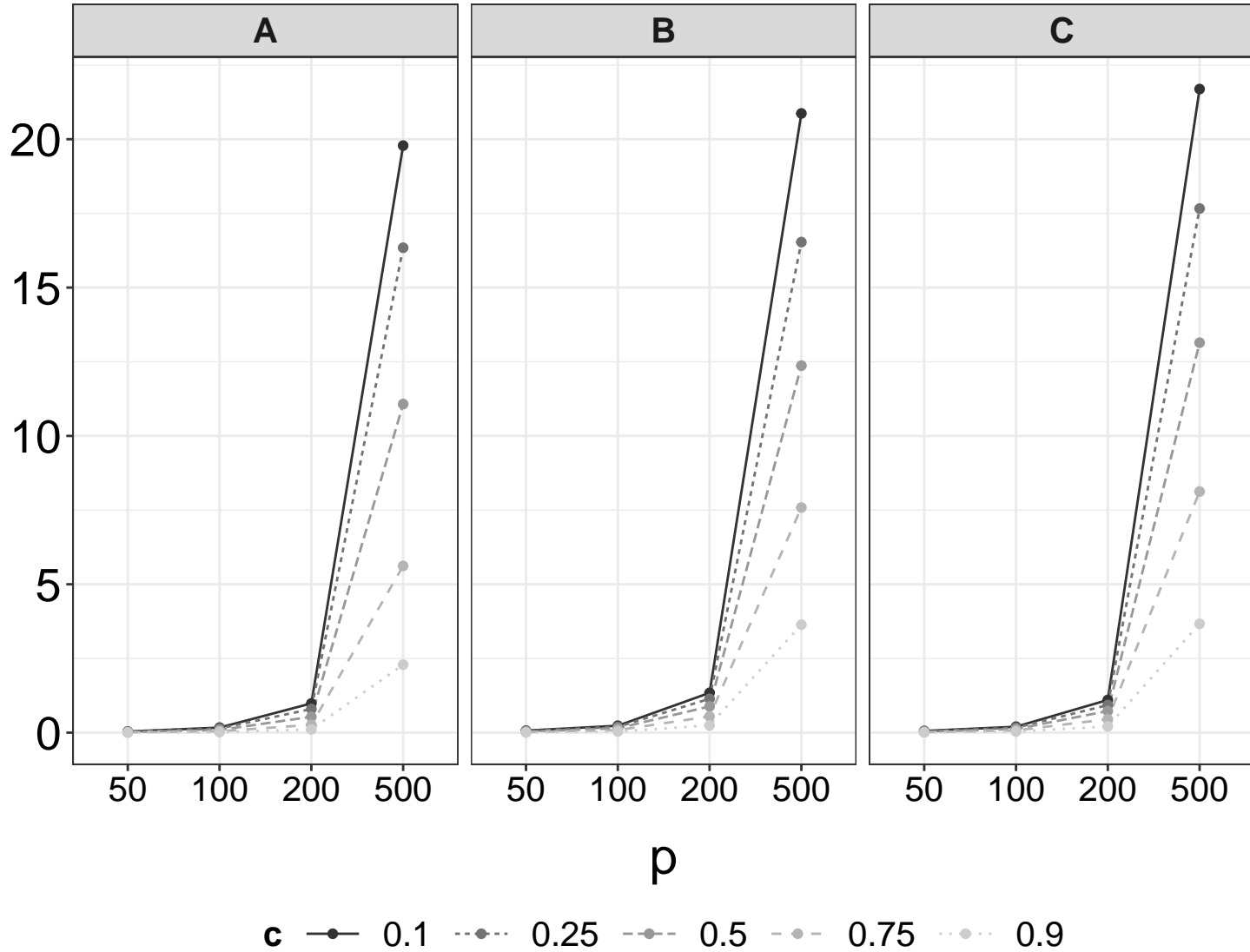

Supplement: Supplementary file 1 — Supporting Information [file BIMJ-66-e70018-s001.zip › KL4GP-Reproducibility/Figures/Fig4_b.pdf]

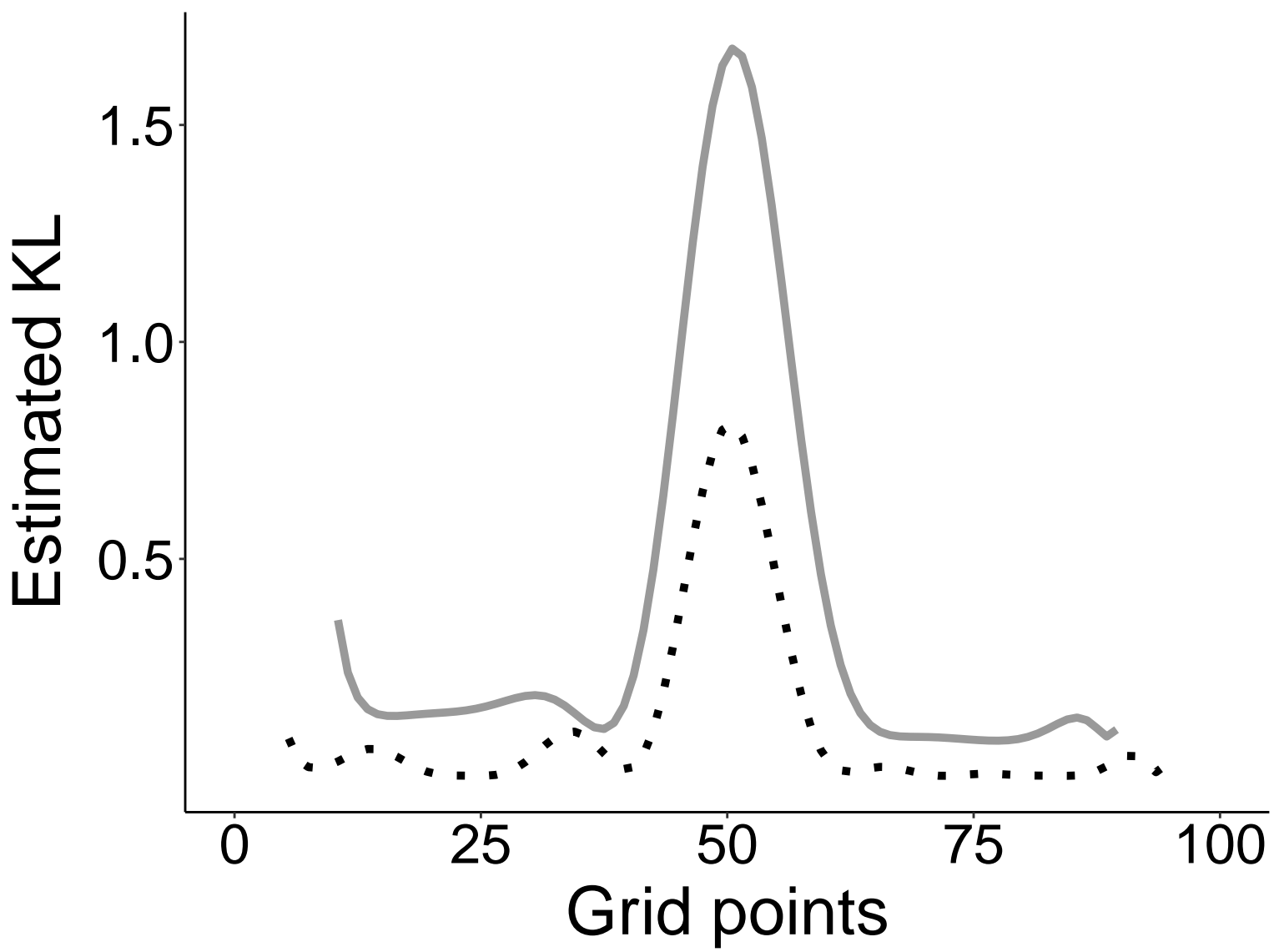

Supplement: Supplementary file 1 — Supporting Information [file BIMJ-66-e70018-s001.zip › KL4GP-Reproducibility/Figures/Fig2_d.pdf]

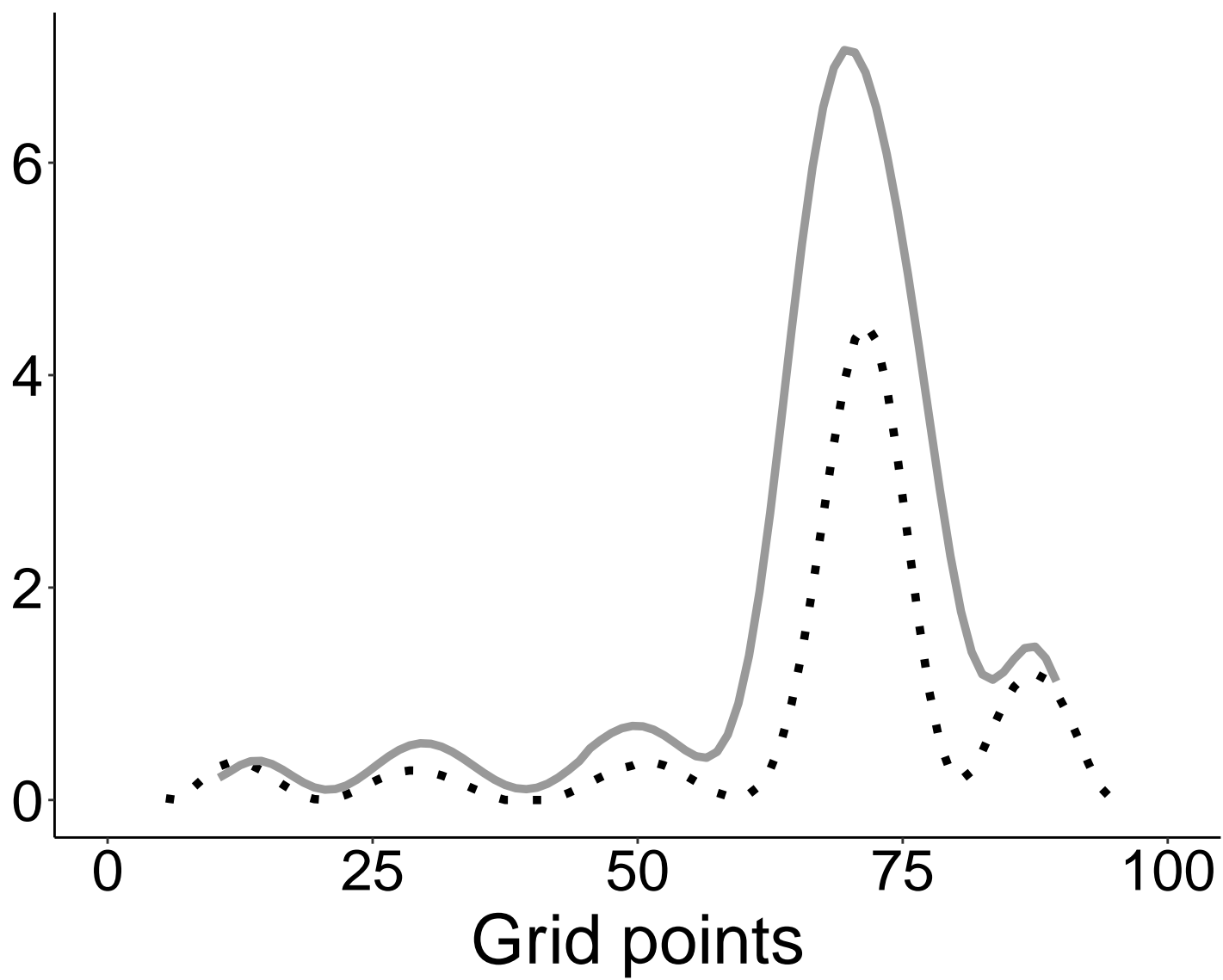

Supplement: Supplementary file 1 — Supporting Information [file BIMJ-66-e70018-s001.zip › KL4GP-Reproducibility/Figures/Fig2_e.pdf]

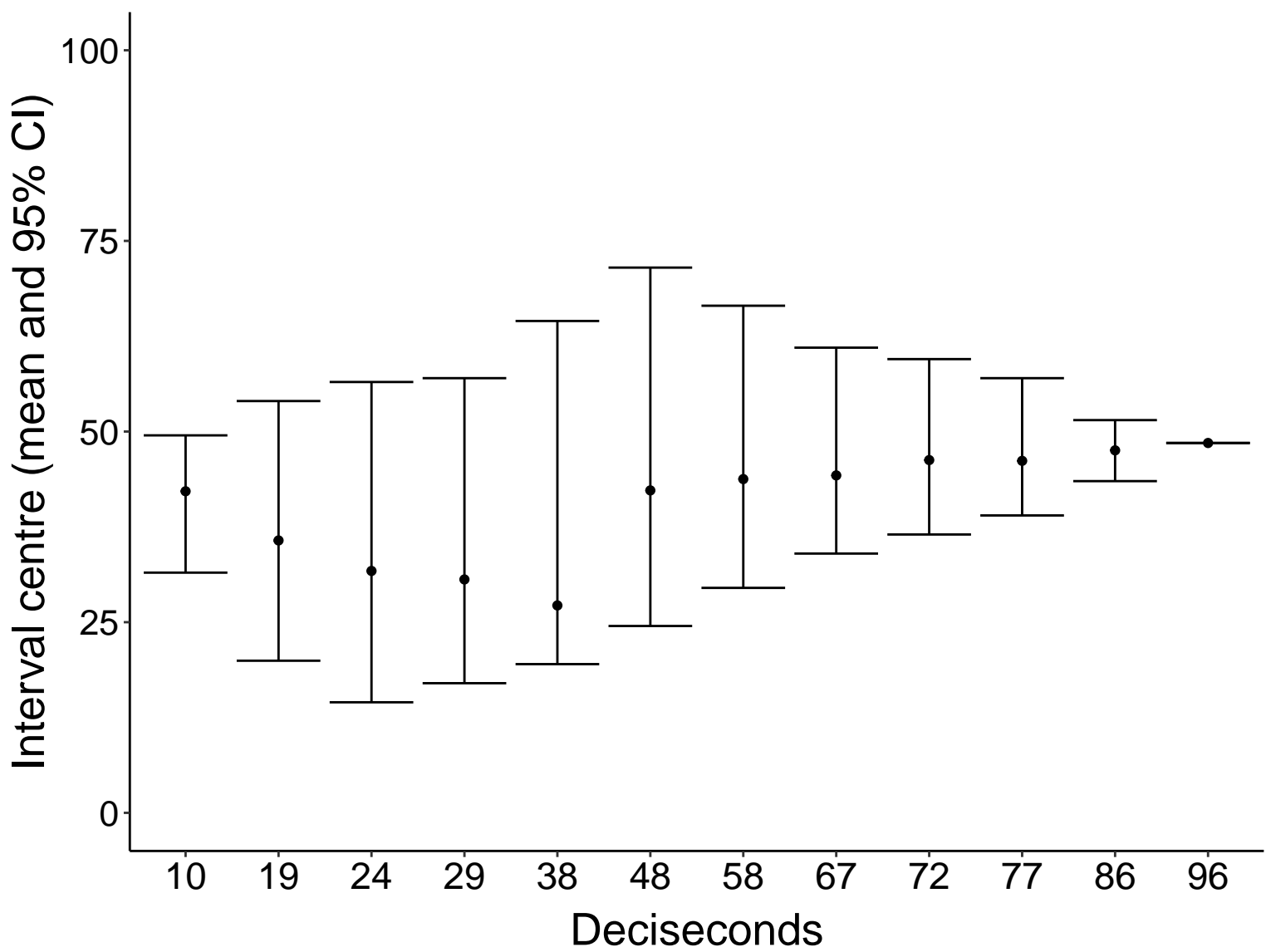

Supplement: Supplementary file 1 — Supporting Information [file BIMJ-66-e70018-s001.zip › KL4GP-Reproducibility/Figures/Fig6_a.pdf]

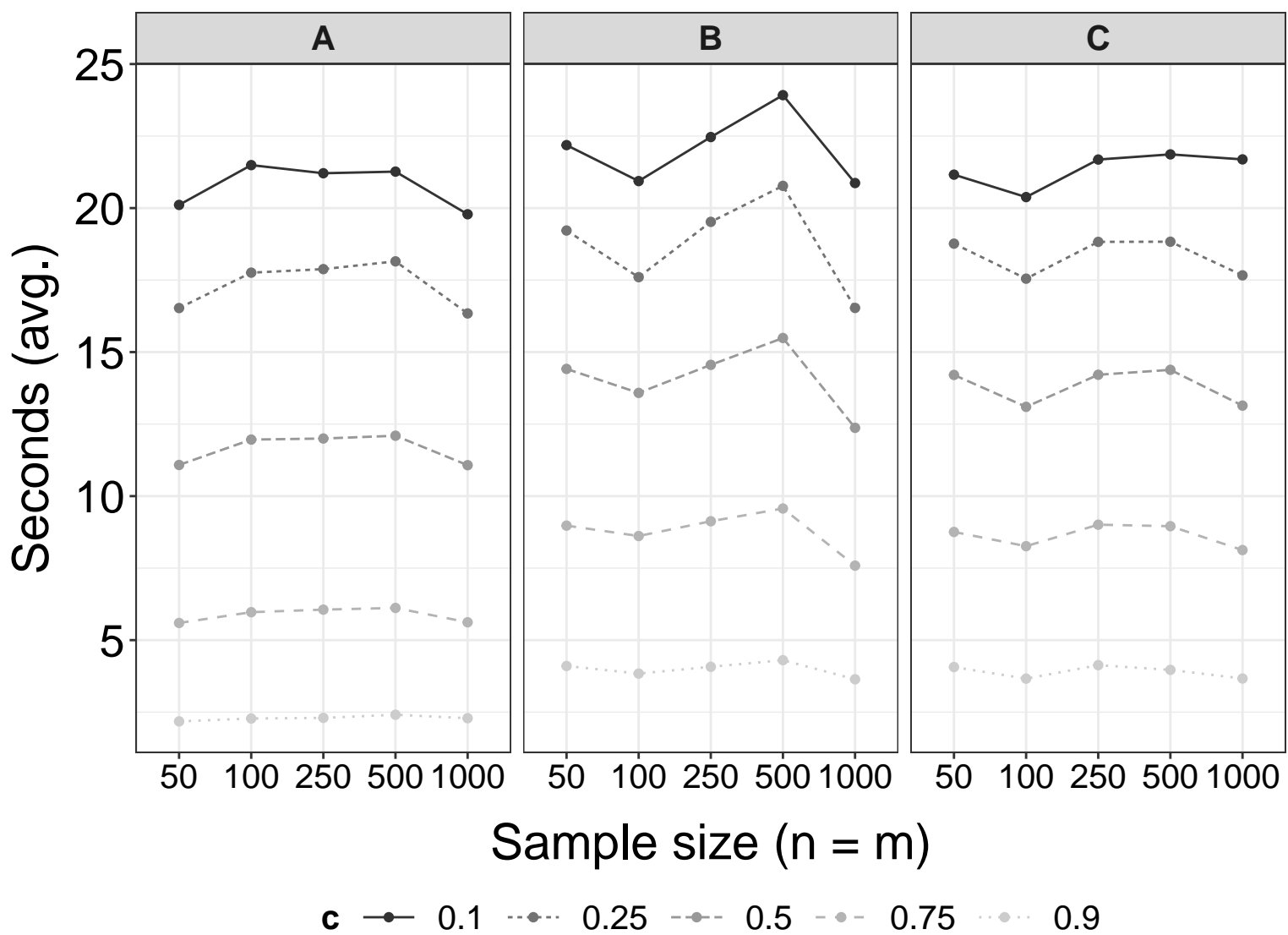

Supplement: Supplementary file 1 — Supporting Information [file BIMJ-66-e70018-s001.zip › KL4GP-Reproducibility/Figures/Fig4_a.pdf]

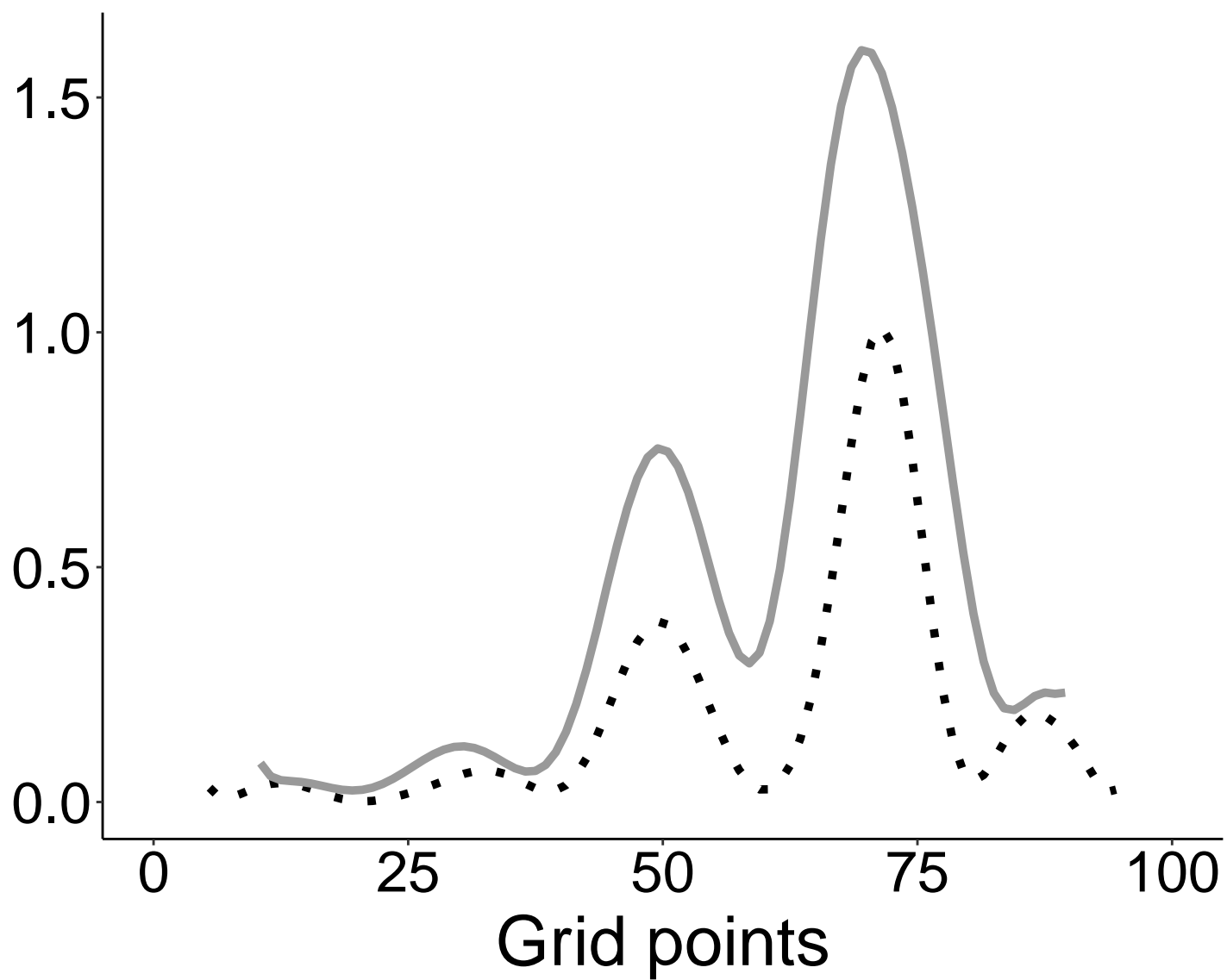

Supplement: Supplementary file 1 — Supporting Information [file BIMJ-66-e70018-s001.zip › KL4GP-Reproducibility/Figures/Fig2_f.pdf]

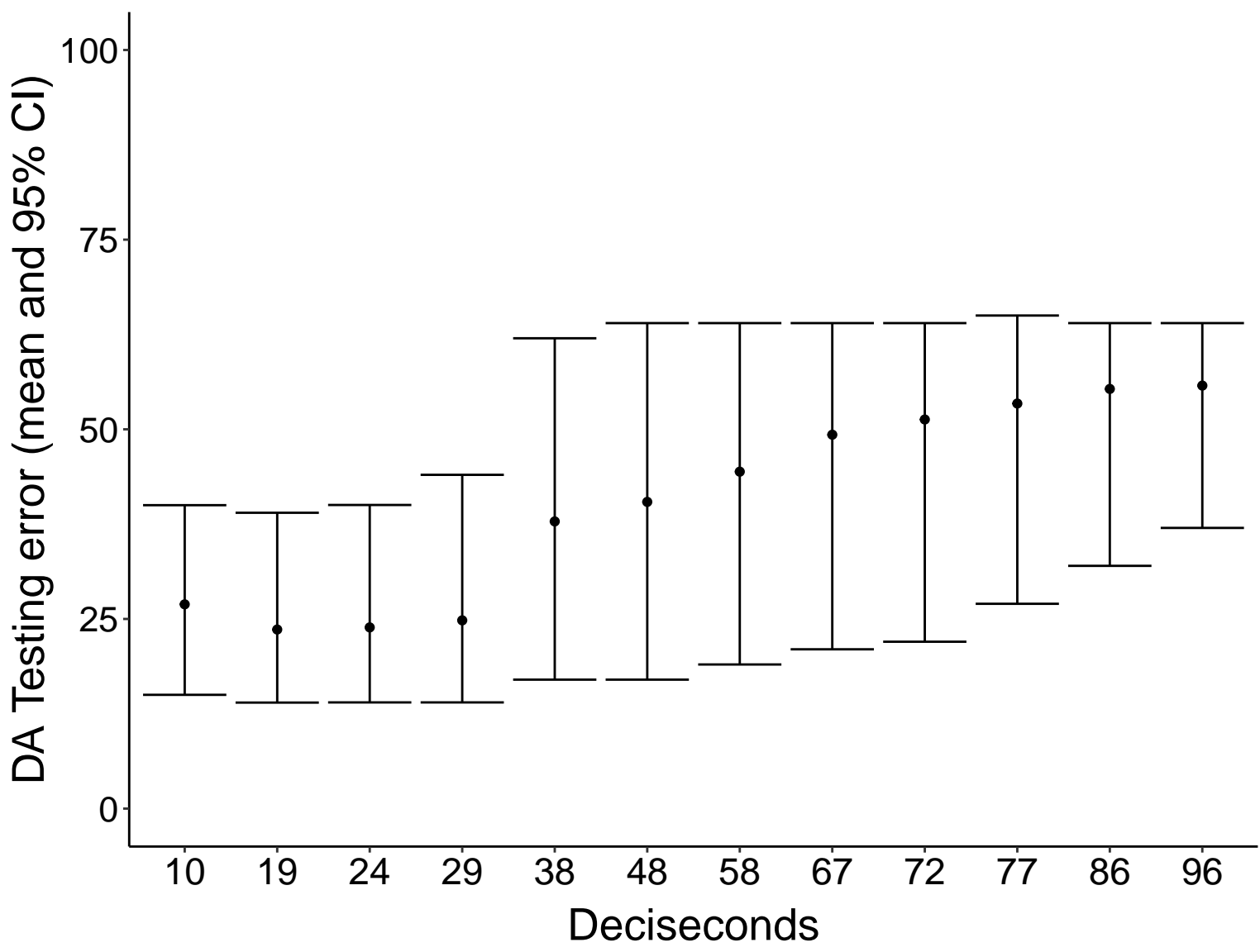

Supplement: Supplementary file 1 — Supporting Information [file BIMJ-66-e70018-s001.zip › KL4GP-Reproducibility/Figures/Fig6_b.pdf]
